# Regulatory mechanisms of autophagy on DHA and carotenoid accumulation in *Crypthecodinium* sp. SUN

**DOI:** 10.1186/s13068-024-02493-6

**Published:** 2024-04-02

**Authors:** Yiming Li, Tiantian Zhao, Weizheng Gao, Bowen Miao, Zhongxiang Fu, Zhao Zhang, Qingyang Li, Dongzhe Sun

**Affiliations:** 1 Ministry of Education Key Laboratory of Molecular and Cellular Biology, Hebei Collaborative Innovation Center for Eco-Environment, Hebei Research Center of the Basic Discipline of Cell Biology, College of Life Sciences, Hebei Normal University, Shijiazhuang, 050024 China; 2 School of Life Sciences, Hebei University, Baoding, 071000 China

**Keywords:** *Crypthecodinium*, Autophagy, DHA, Carotenoid, PI3K-AKT signaling pathway

## Abstract

**Background:**

Autophagy is a crucial process of cellular self-destruction and component reutilization that can affect the accumulation of total fatty acids (TFAs) and carotenoids in microalgae. The regulatory effects of autophagy process in a docosahexaenoic acid (DHA) and carotenoids simultaneously producing microalga, *Crypthecodinium* sp. SUN, has not been studied. Thus, the autophagy inhibitor (3-methyladenine (MA)) and activator (rapamycin) were used to regulate autophagy in *Crypthecodinium* sp. SUN.

**Results:**

The inhibition of autophagy by 3-MA was verified by transmission electron microscopy, with fewer autophagy vacuoles observed. Besides, 3-MA reduced the glucose absorption and intracellular acetyl-CoA level, which resulting in the decrease of TFA and DHA levels by 15.83 and 26.73% respectively; Surprisingly, 3-MA increased intracellular reactive oxygen species level but decreased the carotenoids level. Comparative transcriptome analysis showed that the downregulation of the glycolysis, pentose phosphate pathway and tricarboxylic acid cycle may underlie the decrease of acetyl-CoA, NADPH and ATP supply for fatty acid biosynthesis; the downregulation of *PSY* and *HMGCR* may underlie the decreased carotenoids level. In addition, the class I PI3K-AKT signaling pathway may be crucial for the regulation of carbon and energy metabolism. At last, rapamycin was used to activate autophagy, which significantly enhanced the cell growth and TFA level and eventually resulted in 1.70-fold increase in DHA content.

**Conclusions:**

Our findings indicate the mechanisms of autophagy in *Crypthecodinium* sp. SUN and highlight a way to manipulate cell metabolism by regulating autophagy. Overall, this study provides valuable insights to guide further research on autophagy-regulated TFA and carotenoids accumulation in *Crypthecodinium* sp. SUN.

**Supplementary Information:**

The online version contains supplementary material available at 10.1186/s13068-024-02493-6.

## Background

Microalgae exist mainly in unicellular or multicellular forms in various environments [1]. Due to their strong adaptability to the environment, microalgae are widely distributed across the land, oceans, and freshwater lakes [2]. Lipids and carotenoids are crucial bioactive substances in microalgae that have been widely used in aquatic products, foods, medicines, and cosmetics [2–4]. *Crypthecodinium* sp. SUN is a newly isolated microalga that has been considered valuable for simultaneous production of fatty acids (including docosahexaenoic acid (DHA) and carotenoids [5, 6]. As a strict heterotrophic microalga, light has been shown to significantly increase its total fatty acid (TFA) and carotenoid levels [5, 6]. An elevated TFA level was accompanied by a decline in the starch level in *C*. sp. SUN [5].

Blocking key enzymes in the starch biosynthesis pathway diverts more carbon flow to lipid biosynthesis in *Chlamydomonas reinhardtii* [7]. Moreover, a starch-deficient *Chlorella sorokiniana* mutant exhibited an increased lipid level [8]. These studies indicate that a decrease in the starch level increases lipid accumulation in some microalgae. Our recent study in *C*. sp. SUN showed that the supply of different precursors might also lead to different patterns of accumulation of storage compounds [9]. These studies suggest that the relationship between starch and fatty acids is more complicated than simple competition for carbon sources and energy. Furthermore, the accumulation of fatty acids and carotenoids under stress conditions was generally accompanied by increased intracellular reactive oxygen species (ROS) in microalgae [10, 11]. However, research on *C*. sp. SUN showed that ROS was significantly decreased with an increase in the carotenoid level in the light [6]. These results indicate a novel regulatory mechanism underlying the accumulation of fatty acids and carotenoids in *C*. sp. SUN.

Autophagy is a self-protective process that widely exists in eukaryotes, such as yeast, mammals, plants, and microalga [12, 13]. Soluble protein, macromolecular substances, and damaged organelles can be degraded by autophagy to provide basic materials for various biosynthetic processes [14]. Compared to yeast, mammals, and plants, autophagy has not been deeply studied in microalgae. Recent studies in microalgae have shown that autophagy can be induced under various stress conditions, which is accompanied by the transformation of protein/starch to lipid [13, 15]. Thus, it is reasonable to speculate that autophagy may participate in starch degradation and increased fatty acid accumulation in *C*. sp. SUN [5]. In addition, intracellular ROS, which is a key stimulator of carotenoid accumulation in microalgae, can be quenched by autophagy [12, 16] and preventing autophagy has been shown to increase carotenoid accumulation by increasing ROS in microalgae [17, 18]. 3-Methyladenine (MA), a specific class III phosphatidylinositol 3-kinase (PI3K) inhibitor that inhibits autophagy, increased intracellular ROS in C*hlorella zofingiensis*, accompanied by astaxanthin accumulation [17]. Additionally, 3-MA not only enhanced intracellular ROS, but also increased TFA and astaxanthin accumulation in *Haematococcus pluvialis* under high light stress [18]. Therefore, autophagy may also regulate the accumulation of fatty acids and carotenoids in *C*. sp. SUN.

Autophagy is mediated by proteins encoded by autophagy-related genes (ATGs). More than 40 ATGs have been reported in yeasts, plants, algae, and metazoans [19]. ATGs play an important role in the various stages of autophagy, including initiation, nucleation, fusion, and degradation [20]. Autophagy has been proposed to be regulated by multiple signaling pathways, including class I PI3K-AKT (phosphatidylinositol 3-kinase and protein kinase B), TOR (target of rapamycin), class III PI3K, and AMPK (adenosine 5‘-monophosphate (AMP)-activated protein kinase) signaling pathways [21–24]. The class I PI3K-AKT signaling pathway may modulate several downstream metabolism pathways, and is related to cell growth, proliferation, and autophagy progression [22, 25]. PI3K activation recruits AKT to the plasma membrane; once AKT is activated by phosphorylation, a number of downstream targets including TOR are phosphorylated and regulated, so as to suppress autophagy, on the contrary, negative regulate TOR could activate autophagy [26]. For instance, the TOR inhibitor rapamycin combines with TORC1 (a TOR complex) and then promotes the activity of autophagy-inducing protein ATG1 (also named ULK1) [24, 27]. Additionally, AMPK, a serine/threonine kinase, can be activated under glucose starvation or a low AMP/ATP ratio, and then catabolic pathways are stimulated to increase ATP production, such as starch degradation by autophagy [28]. The relationship between autophagy and the abovementioned signaling pathways have been well established in yeasts, plants, and mammals [21, 26]. However, the mechanisms of autophagy and the related signaling pathways in microalgae remain unclear [21, 24, 26].

In this study, the autophagy inhibitor 3-MA was used to explore the regulatory mechanisms underlying the accumulation of fatty acids and carotenoids in *C*. sp. SUN. The ultrastructure of *C*. sp. SUN was observed by transmission electron microscopy (TEM) to verify that 3-MA inhibited autophagy. Next, the cell number, dry weight (DW), and glucose consumption were measured to study the effects of autophagy on cell growth. To investigate the effects of autophagy on organic carbon distribution, the levels of starch, TFA, protein, DHA, lipid subclasses, and acetyl-CoA were quantified in the 3-MA and control groups. Additionally, intracellular ROS and carotenoid levels were quantified to explore the effects of autophagy on the accumulation of carotenoids in *C*. sp. SUN. Furthermore, comparative transcriptome analysis was conducted to clarify the possible autophagy-related regulatory mechanisms. Finally, rapamycin was employed to verify the role of upregulating autophagy in the production of lipids and carotenoids in *C*. sp. SUN. This study provides new insights for future research on the regulatory mechanisms underlying fatty acid and carotenoid accumulation in *C*. sp. SUN.

## Results and discussion

### Effects of 3-MA on ultrastructure, cell growth and reactive oxygen species in *C*. sp. SUN

**Fig. 1:**
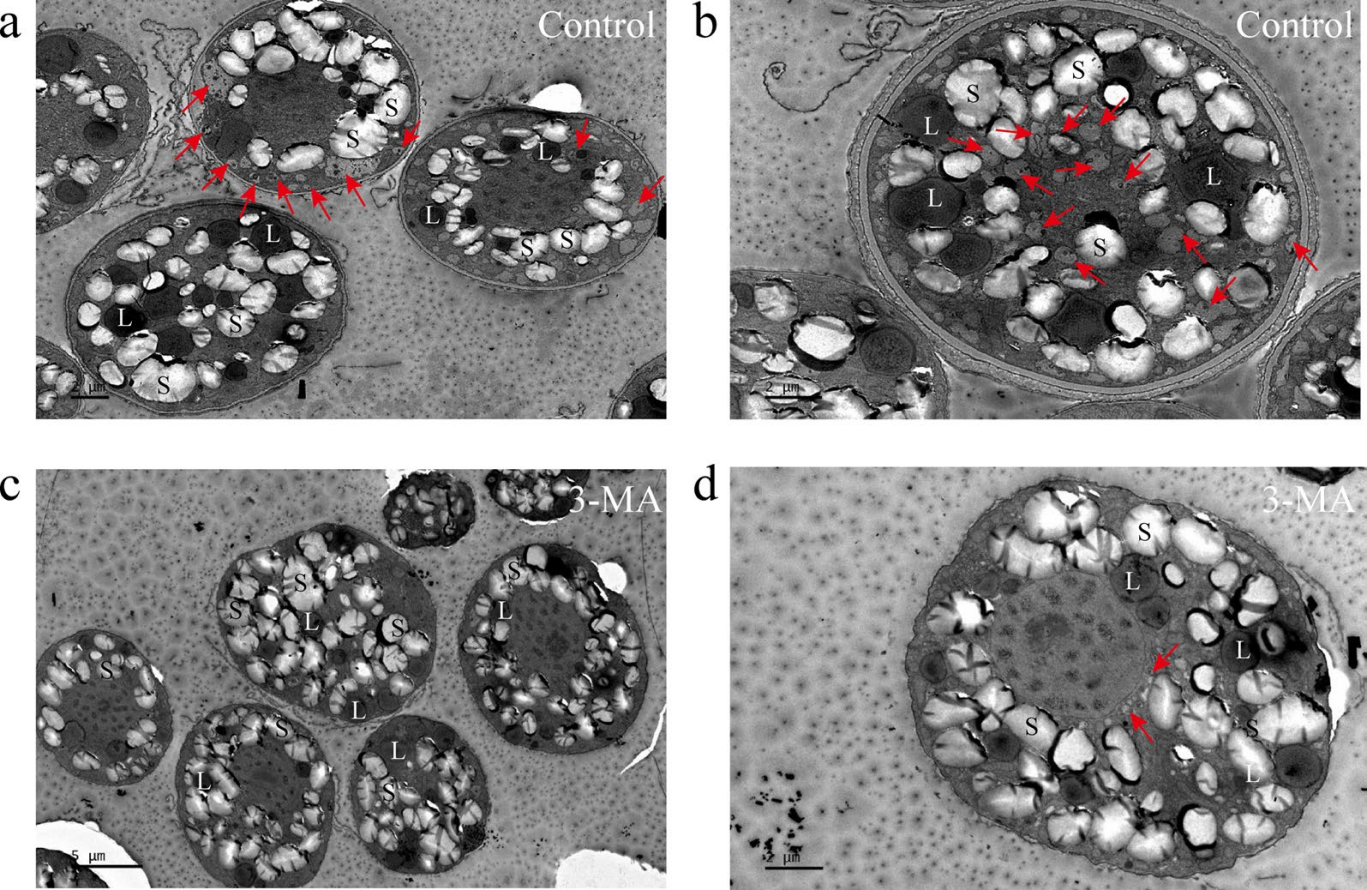
Transmission electron microscopy (TEM) images of *C*. sp. SUN at 48 h. **a**, **b** Control group. **c**, **d** 3-MA group. *S* starch granule, *L* lipid droplet, *red arrowhead* autophagic vacuole

3-MA has been widely used as an autophagy inhibitor in higher plants and microalgae [29–31]. Autophagic activity can be assessed by observing autophagy-related structures [12]. Autophagic vacuoles, as typical ultrastructure components during the autophagic process, have been defined as membrane-bound vacuoles [12]. These vacuoles contain fragments of cellular components, such as mitochondria, endoplasmic reticulum, or chloroplasts [29]. The presence of autophagic vacuoles observed under transmission electron microscopy (TEM) serves as strong evidence for autophagy in higher plants and model microalgae like *Chlamydomonas reinhardtii* [29]. Thus, the TEM was used to verify the presence of autophagic vacuoles (which are typical ultrastructure components during the autophagic process) in order to confirm the inhibitory effects of 3-MA on autophagy in *C*. sp. SUN [30]. Compared to the control group, 3-MA significantly decreased the autophagic vacuoles (red arrows in Fig. 1), indicating that 3-MA inhibited autophagy in *C*. sp. SUN. This result is consistent with research showing that 3-MA inhibited autophagic vacuoles in *Chlorella* NC64A [30].

**Fig. 2:**
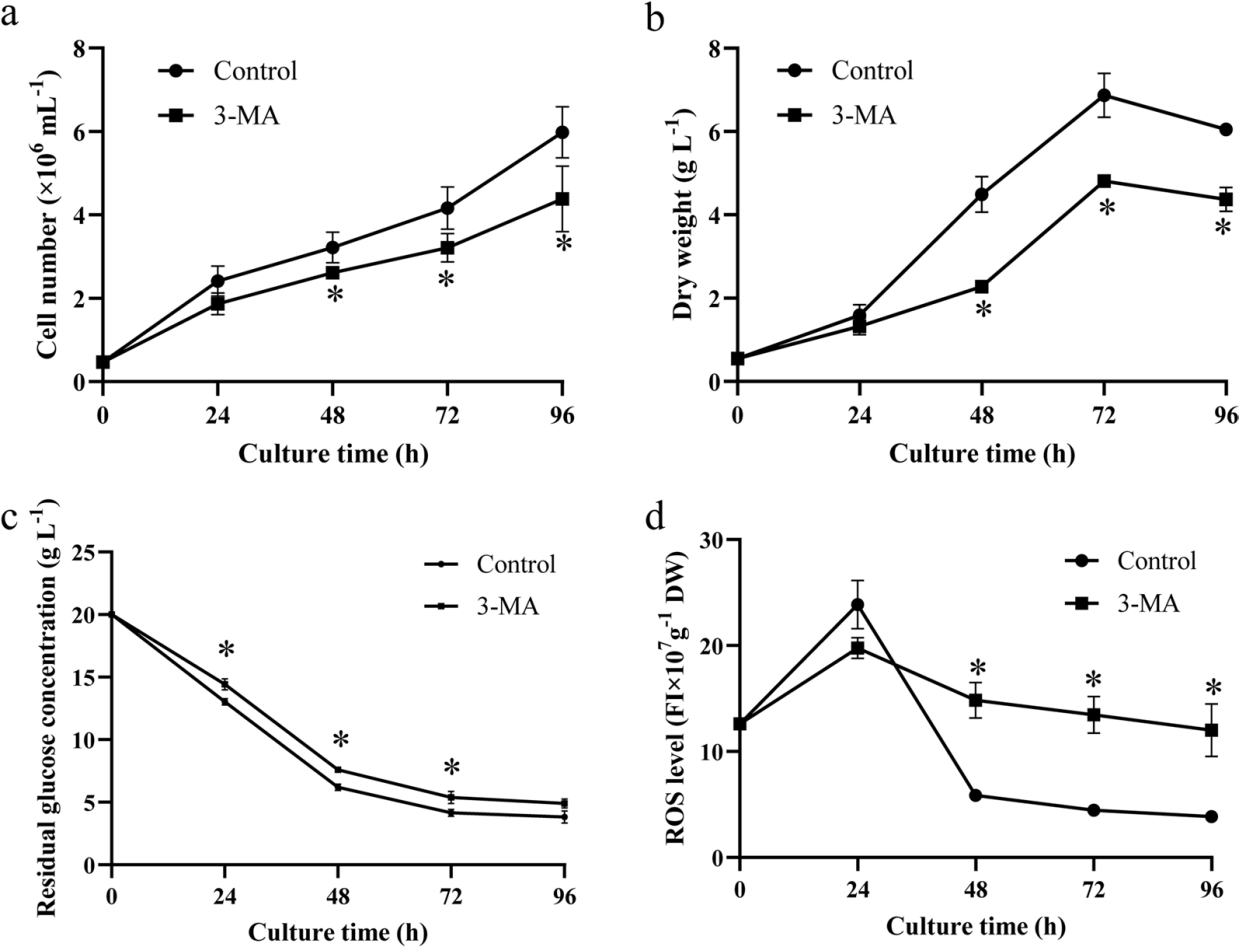
Cell growth, residual glucose concentration, and reactive oxygen species (ROS) in *C*. sp. SUN. **a** Cell number. **b** Dry weight (DW). **c** Residual glucose concentration. **d** Reactive oxygen species (ROS). Three biological replicates were conducted per time point in each group. Data represent mean ± standard deviation. * p < 0.05 vs control group within each time point

Additionally, the cell number and DW during cultivation were measured. The cell number in the control group was always higher than the 3-MA group throughout cultivation, and it peaked (5.98 × 10^6^ mL^−1^) at 96 h, which was 1.37-fold greater than that in the 3-MA group (Fig. 2a). DW exhibited a similar trend to the cell number; the only difference was that it peaked (6.87 g L^−1^) at 72 h in the control group, which was 1.43-fold greater than that in the 3-MA group. These results suggest that the addition of the autophagy inhibitor 3-MA significantly inhibited the cell growth of *C*. sp. SUN.

Glucose is the carbon source used during the cultivation of *C*. sp. SUN. The absorption and utilization of glucose was measured to explore the reason underlying the difference in cell growth between the two groups. The residual glucose concentration in the culture medium decreased dramatically within 48 h and then gradually stabilized in both groups (Fig. 2c). The residual glucose concentration in the 3-MA group was reduced to 4.90 g L^−1^, which was 1.28-fold greater than that in the control group. These results indicate that inhibition of autophagy reduced glucose absorption and utilization, which might be the reason for the lower cell number and DW in the 3-MA group.

As ROS triggers autophagy under stress conditions in microalgae, and autophagy in turn quenches excessive intracellular ROS [12, 16], intracellular ROS was measured in both groups (Fig. 2d). The ROS level initially increased and then decreased after 24 h in both groups. With the inhibition of autophagy, ROS levels in the 3-MA group were 2.53-, 3.02-, and 3.10-fold greater than the levels in the control group at 48, 72, and 96 h, respectively, indicating that the inhibition of autophagy significantly increased ROS in *C*. sp. SUN. These results are consistent with research in *Haematococcus pluvialis* [18]. Therefore, autophagy might participate in quenching ROS in *C*. sp. SUN. ROS accumulation increases storage substances and carotenoid accumulation in microalgae [10, 32]. For instance, 3-MA increased intracellular ROS and induced astaxanthin accumulation in *Chlorella zofingiensis* [17]. Thus, the storage substances and carotenoid accumulation were assessed in subsequent experiments.

### Effects of 3-MA on starch, TFA, protein, fatty acid profile, DHA, lipid subclasses, and acetyl-CoA in *C*. sp. SUN

**Fig. 3:**
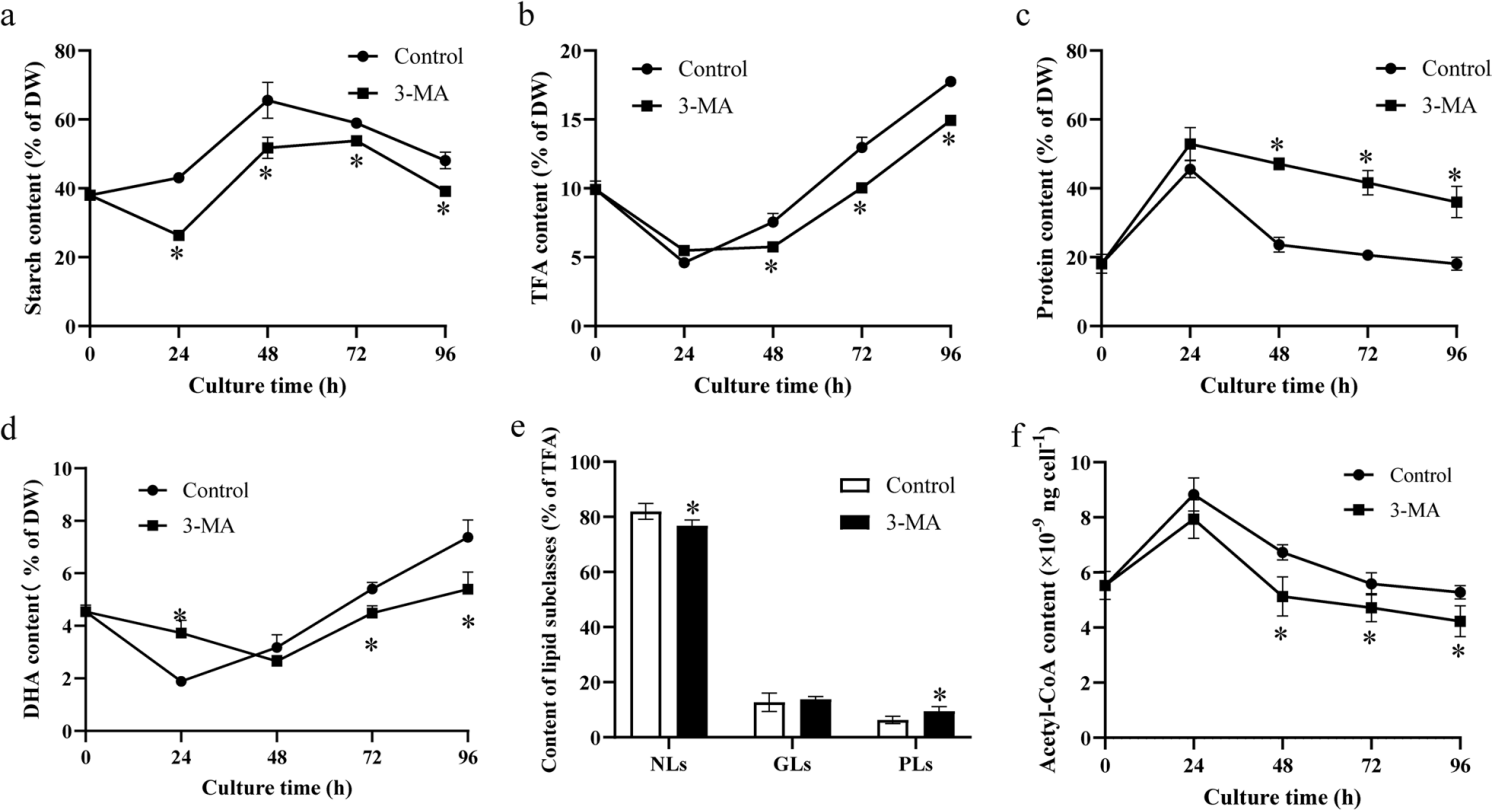
Cell composition, DHA, lipid subclassed, and acetyl-CoA levels in *C*. sp. SUN. **a** Starch. **b** TFA. **c** Protein. **d** DHA. **e** Lipid subclasses. **f** Acetyl-CoA. Three biological replicates were conducted per time point in each group. Data represent mean ± standard deviation. * p < 0.05 vs control group within each time point

To investigate the effects of autophagy on organic carbon distribution, the starch, TFA, and protein levels in the two groups were quantified. The starch level in the control group rapidly increased from 24 to 48 h and peaked (65.59% of DW) at 48 h, which was 1.27-fold greater than that in the 3-MA group (Fig. 3a). After 48 h of cultivation, the starch level gradually decreased in both groups. The TFA levels in the two groups gradually increased after 24 h of cultivation (Fig. 3b). The TFA levels in the 3-MA group peaked (14.94% of DW) at 96 h, which decreased by 15.83% compared to the control group. These results suggest that the inhibition of autophagy decreased starch and TFA accumulation in *C*. sp. SUN. This phenomenon is different from the results in other microalgae. In *Haematococcus pluvialis*, 3-MA significantly increased the TFA level [18]. These results indicate that special carbon metabolism mechanisms might exist in *C*. sp. SUN. Besides starch and fatty acids, protein is another major substance in cells. Due to the lower starch and TFA levels in the 3-MA group, the protein level was significantly higher in the 3-MA group than the control group (Fig. 3c), indicating that the inhibition of autophagy increased protein accumulation in *C*. sp. SUN.

3-MA not only affected the TFA level, but also had significant effects on fatty acid profiles. The proportions of saturated fatty acids (SFAs) were 48.42 and 52.79% of TFA at 24 and 48 h in the control group, respectively, which were 1.18- and 1.11-fold greater than the levels in the 3-MA group (Table 1). These results indicate that the inhibition of autophagy significantly decreased the proportion of SFAs in *C*. sp. SUN. The percentage of monounsaturated fatty acids (MUFAs) in the control group gradually decreased from 0 to 96 h and was lowest (12.35% of TFA) at 96 h, which was 1.13-fold greater than that in the 3-MA group. However, unlike SFAs and MUFAs, the proportion of polyunsaturated fatty acids (PUFAs) were significantly higher in the 3-MA group than the control group. Specifically, the PUFAs accounted for 46.64% and 40.71% of TFA at 24 and 48 h, respectively, which were 1.20- and 1.19-fold greater than that in the control group. The percentage of DHA peaked (45.60% of TFA) at 24 h in the 3-MA group, which was 1.23-fold greater than that in the control group. After 24 h of cultivation, the DHA level gradually decreased. Thus, 3-MA significantly decreased the SFA and MUFA levels, but increased the PUFA level (including DHA) in *C*. sp. SUN. These results indicate that *C*. sp. SUN tends to accumulate PUFA when autophagy is suppressed, which are consistent with results in *Chlorella zofingiensis* [17]. However, the change in DHA as a % of TFA did not mirror the change in DHA as a % of DW. The latter measure of DHA initially decreased and then increased after 48 h in both groups (Fig. 3d). It peaked (5.40% of DW) in the 3-MA group at 96 h, which decreased by 26.73% compared to the control group. This result indicates that the inhibition of 3-MA significantly decreased DHA as a % of DW in *C*. sp. SUN.

**Table 1:** Fatty acid profiles (% of TFA) in *C*. sp. SUN

Time (h)	Group	C14:0	C16:0	C18:0	C18:1	C22:6	SFAs	MUFAs	PUFAs
0	Control	18.42 ± 0.55	21.68 ± 0.59	2.95 ± 0.12	12.04 ± 0.12	38.77 ± 1.33	47.81 ± 1.45	12.64 ± 0.12	39.56 ± 1.41
24	Control	16.34 ± 0.22	26.50 ± 0.65	2. 85 ± 0.33	11.76 ± 0.18	37.11 ± 1.44	48.42 ± 1.36	12.84 ± 0.30	38.74 ± 1.27
	3-MA	15.21 ± 1.97	22.19 ± 0.63^*^	1.80 ± 0.24^*^	11.74 ± 0.40	45.60 ± 2.16^*^	40.93 ± 2.77^*^	12.43 ± 0.62	46.64 ± 2.19^*^
48	Control	19.29 ± 0.47	26.54 ± 0.29	4.18 ± 0.41	12.08 ± 0.36	33.17 ± 1.09	52.79 ± 0.96	12.91 ± 0.42	34.30 ± 0.42
	3-MA	16.10 ± 0.38^*^	26.21 ± 0.57	3.20 ± 0.82^*^	10.45 ± 0.23^*^	37.60 ± 0.93^*^	47.75 ± 2.52^*^	11.54 ± 0.48^*^	40.71 ± 0.99^*^
72	Control	19.46 ± 1.19	23.84 ± 1.08	4.46 ± 0.38	11.72 ± 0.21	36.21 ± 2.44	49.98 ± 2.85	12.52 ± 0.28	37.50 ± 2.98
	3-MA	18.95 ± 0.52^*^	24.15 ± 0.14	4.39 ± 0.10	10.46 ± 0.26	35.76 ± 0.33	49.92 ± 0.70	11.28 ± 0.34	38.79 ± 0.63
96	Control	17.69 ± 0.26	22.35 ± 0.50	4.13 ± 0.18	11.41 ± 0.17	38.77 ± 0.75	46.48 ± 0.48	12.35 ± 0.18	41.17 ± 0.55
	3-MA	18.38 ± 1.21	23.92 ± 1.08	3.75 ± 0.25	10.15 ± 0.27	38.23 ± 1.62	48.64 ± 2.49^*^	10.96 ± 0.29^*^	40.41 ± 2.65

C14:0, myristic acid; C16:0, palmitic acid; C18:0, stearic acid; C18:1, oleic acid; C22:6, docosahexaenoic acid. Three biological replicates were conducted per time point in each group. Data represent mean ± standard deviation. * p < 0.05 vs control group within each time point

*SFAs* saturated fatty acids as a % of total fatty acids (TFA), *MUFAs* monounsaturated fatty acids as a % of TFA, *PUFAs* polyunsaturated fatty acids as a % of TFA

Total lipids in *C*. sp. SUN at 96 h were separated into neutral lipids (NLs), glycolipids (GLs), and phospholipids (PLs). NLs in the control group accounted for 80.99% of TFA, which was 1.06-fold greater than that in the 3-MA group (Fig. 3e). PLs in the 3-MA group accounted for 9.48% of TFA, which was 1.50-fold greater than that in the control group. 3-MA had no significant effect on GL compared to the level in the control group. These results indicate that the inhibition of autophagy significantly decreased NLs (the main storage lipids in microalgae [5]) but increased PLs (PLs are closely related to membrane fluidity in microalgae [33, 34]), both as a % of TFA, in *C*. sp. SUN. The inhibition of autophagy might decrease organelle degradation and then maintain more membrane structures in cells. Thus, the inhibition of autophagy may underlie the increase in PLs as a % of TFA in *C*. sp. SUN in the 3-MA group.

To explore the reason underlying the lower TFA level in the 3-MA group compared to the control group, the level of acetyl-CoA (precursor for fatty acid biosynthesis) in the two groups was quantified (Fig. 3f). In both groups, the acetyl-CoA level initially increased and then started to decrease after 24 h. Compared to the control group, the acetyl-CoA levels in the 3-MA group decreased by 10.08, 23.75, 15.54, and 19.77% at 24, 48, 72, and 96 h, respectively. These results indicate that the inhibition of autophagy significantly decreased the intracellular acetyl-CoA level, which may underlie the lower TFA level in the 3-MA group (Fig. 3b).

### Effects of 3-MA on carotenoid level in *C*. sp. SUN

**Fig. 4:**
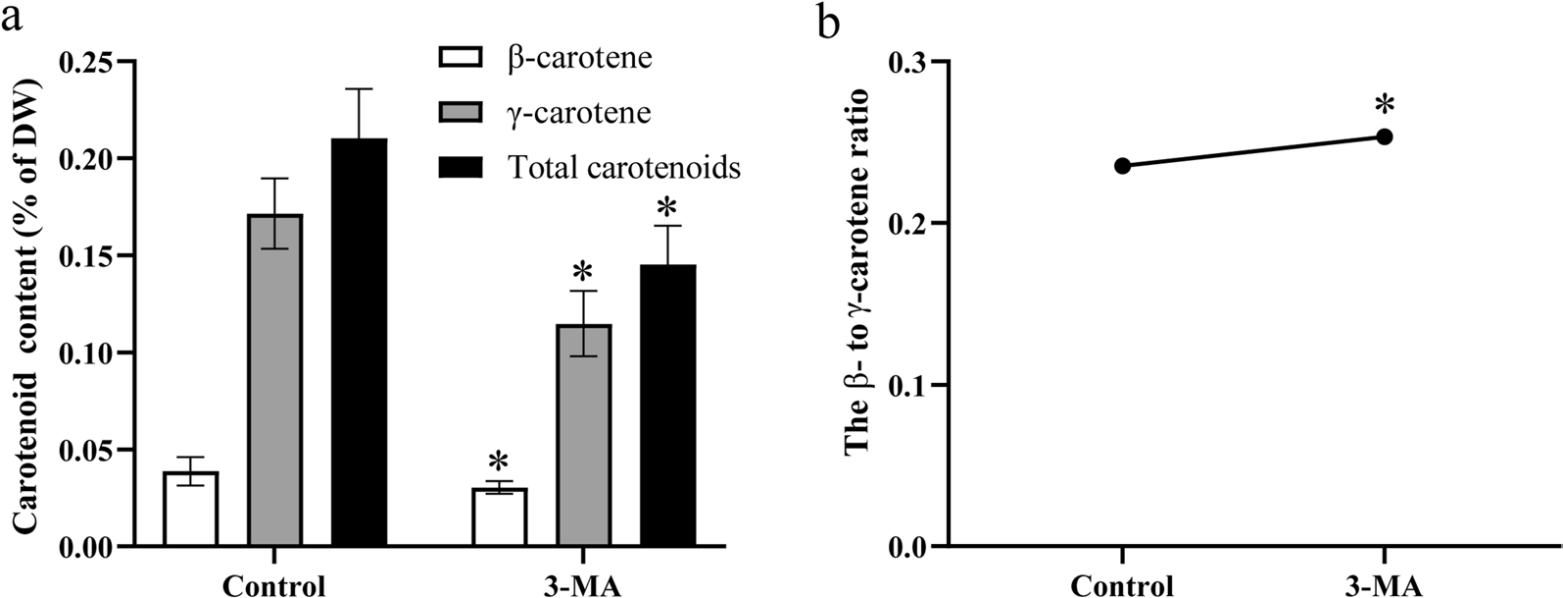
Carotenoid level and β- to γ-carotene ratio in *C*. sp. SUN at 96 h. **a** Carotenoid level. **b** β- to γ-carotene ratio. Three biological replicates were conducted in each group. Data represent mean ± standard deviation. * p < 0.05 vs control group within each time point

Autophagy has been found to affect carotenoid accumulation through intracellular ROS levels in microalgae [17, 35]. However, the effects of autophagy on the carotenoid level in *C*. sp. SUN still remain unclear. Thus, after cultivation for 96 h, the carotenoids were extracted and quantified. There were only two kinds of carotenoids in *C*. sp. SUN: β- and γ-carotene. 3-MA significantly reduced both carotenoids (Fig. 4a). The total carotenoid level in the control group was 0.21% of DW, which was 1.45-fold greater than that in the 3-MA group, suggesting that the inhibition of autophagy significantly decreased the carotenoid level. However, compared to the control group, 3-MA significantly increased the β- to γ-carotene ratio (Fig. 4b), indicating that the inhibition of autophagy converted more γ- to β-carotene in *C*. sp. SUN. 3-MA significantly increased intracellular ROS (Fig. 2d), while decreasing the carotenoid level (Fig. 4). The relationship between ROS and carotenoid levels was consistent with the results of a previous study in *C*. sp. SUN, where ROS was decreased but the total carotenoid level was increased by more than 10 times under high light conditions (100 μmol photons m^−2^ s^−1^) [6]. However, the findings are different from those related to other microalgae under stress conditions, in which ROS and carotenoid levels were elevated simultaneously [17]. These results suggest that the carotenoid regulatory mechanism might be more complex in *C*. sp. SUN. As inhibition of autophagy decreased both fatty acid and carotenoid accumulation, increasing autophagy may be a method to increase the accumulation of fatty acids and carotenoids simultaneously in *C*. sp. SUN.

### Comparative transcriptome analysis

**Fig. 5:**
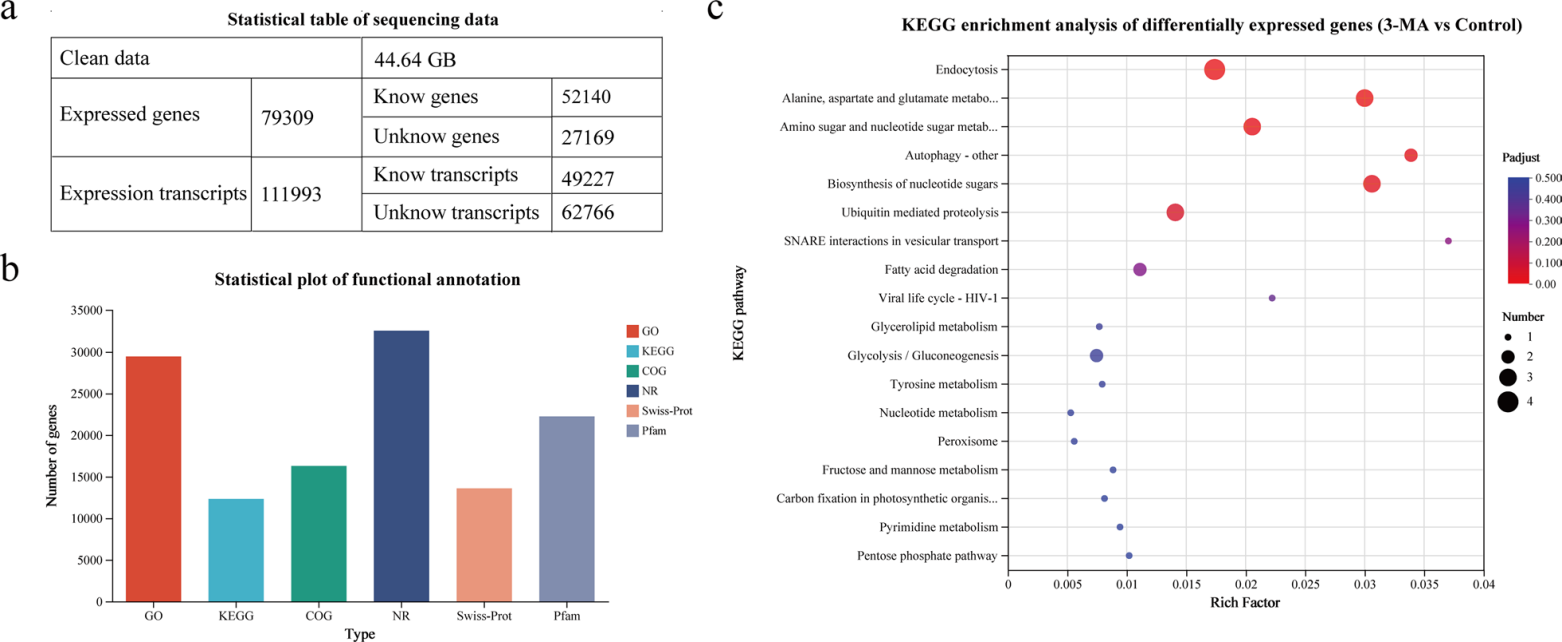
Overview of comparative transcriptome analysis in *C*. sp. SUN. **a** Sequencing statistics. **b** Bar chart of number of annotated genes by database (GO, KEGG, COG, NR, Swiss-Port and Pfam). **c** KEGG enrichment analysis of differentially expressed genes between the 3-MA and control groups

To explore the regulatory mechanisms underlying the effects of 3-MA on organic carbon distribution and carotenoid accumulation, a comparative transcriptome analysis was conducted after 48 h of culture of *C*. sp. SUN. The raw sequence data were deposited in the Genome Sequence Archive (CRA011097) at the National Genomics Data Center. RNA-seq detected 79,309 expressed genes (52,140 were known and 27,169 were new) and 111,993 transcripts (49,227 were known and 62,766 were new) (Fig. 5a). The expressed genes and transcripts were annotated using functional databases (NR, GO, COG, KEGG, Swiss-Port, and Pfam) (Fig. 5b).

In addition, a KEGG enrichment analysis of the significant differentially expressed genes between the 3-MA and control groups was conducted Fig. 5c. The “Endocytosis” and “Autophagy” pathways were enriched, which indicates that the autophagy process in *C*. sp. SUN was significantly affected by 3-MA. Additionally, the enrichment of “Fatty acid degradation”, “Glyceride metabolism” and “Fructose and mannose metabolism” indicates that organic carbon metabolism was significantly different between the two groups. As crucial pathways for acetyl-CoA, ATP and NADPH supply, the glycolysis pathway, tricarboxylic acid (TCA) cycle, and pentose phosphate pathway were also studied, among which the glycolysis and pentose phosphate pathways were enriched. Details regarding the genes in the above pathways are summarized in Additional file 1: Table S1. The key genes in the various metabolic pathways with transcripts per million reads (TPM) > 10 were selected and are detailed in the following sections.

**Fig. 6:**
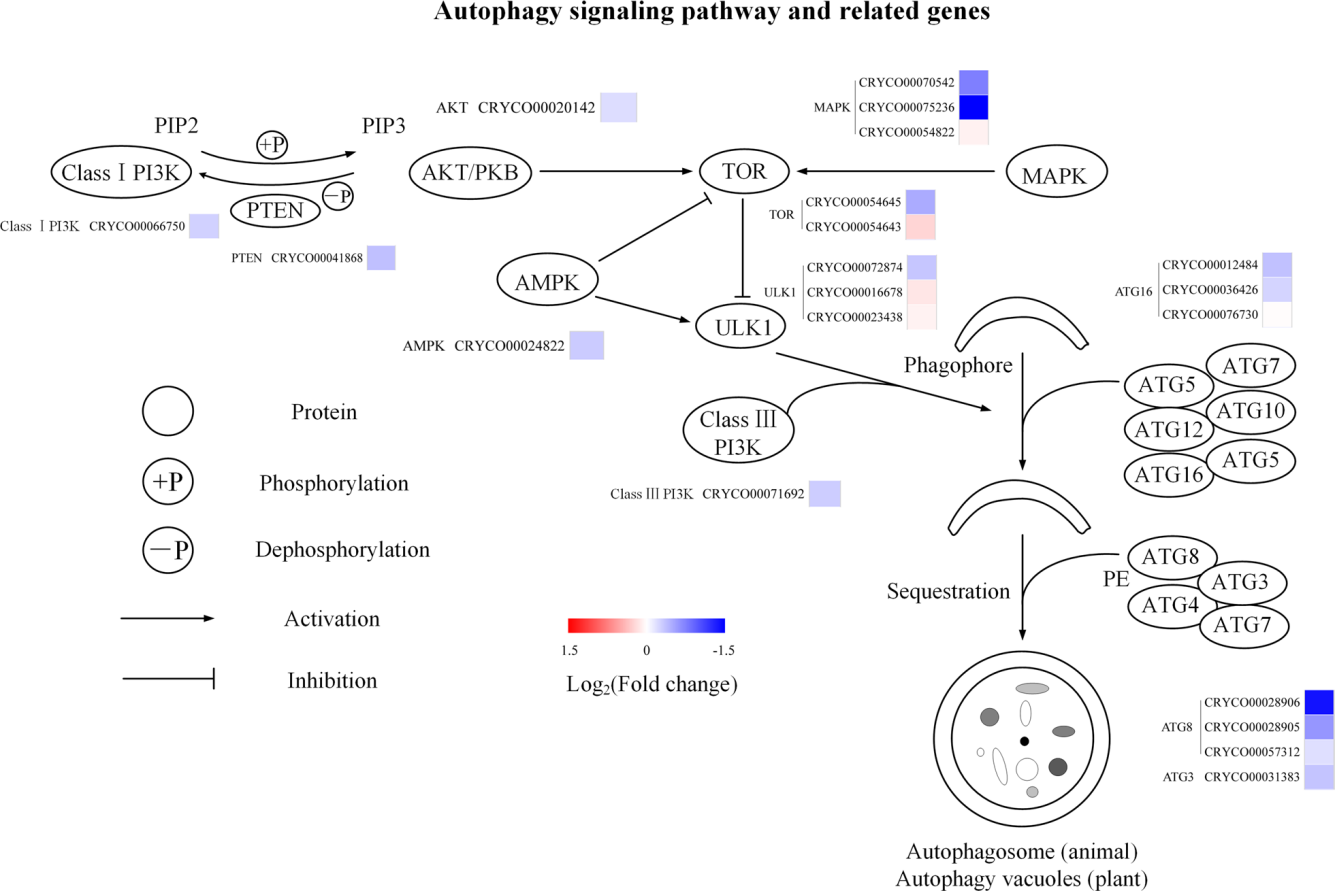
Expression of genes in autophagy in *C*. sp. SUN after 48 h. Red (upregulated) and blue (downregulated) log_2_(fold change) values represent the expression in the 3-MA group compared to the control group. See Additional file 1: Table S1 for more details on the RNA-seq data

**Fig. 7:**
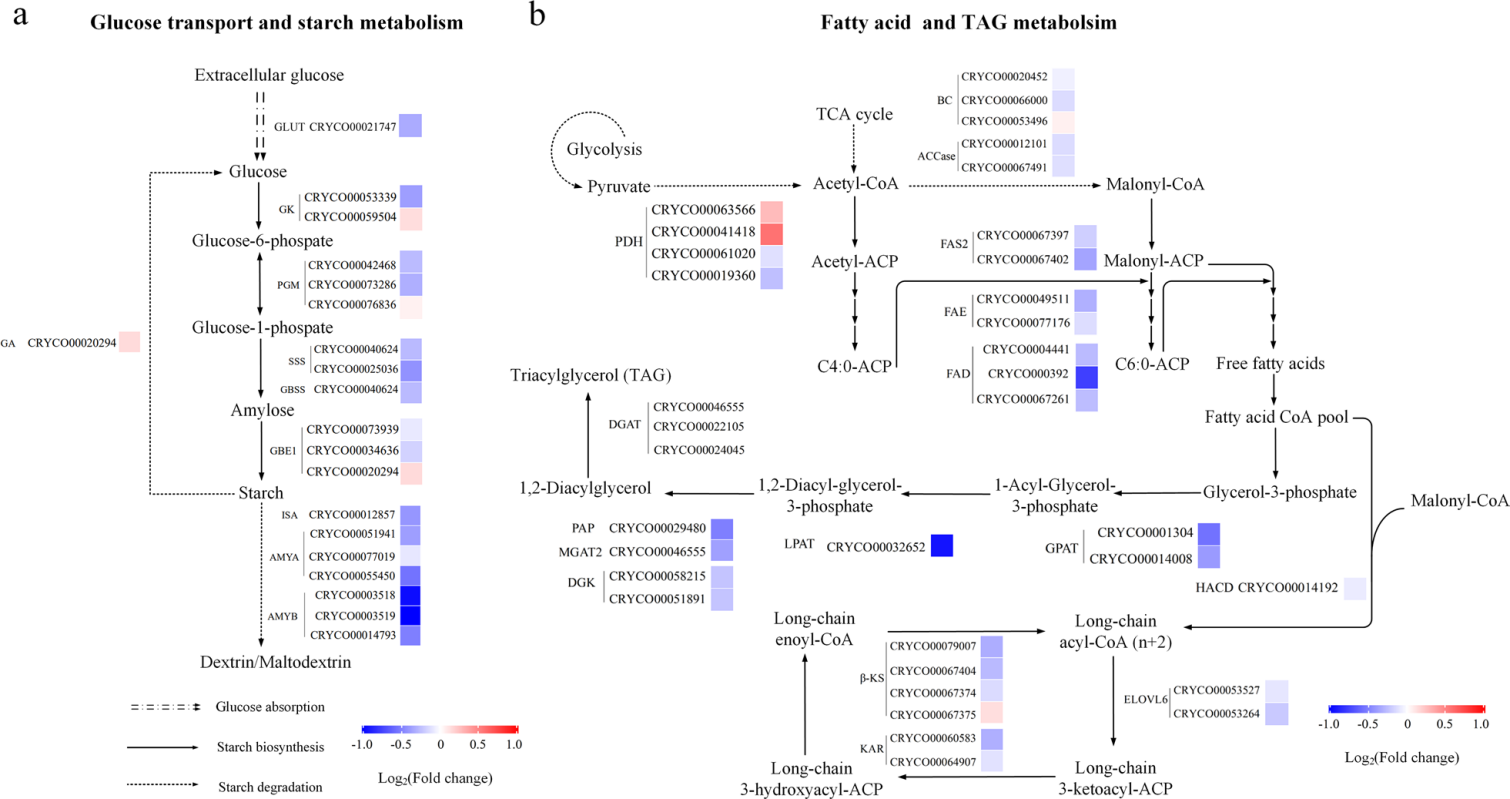
Expression of genes in starch and lipid metabolism in *C*. sp. SUN after 48 h. **a** Glucose transport and starch metabolism. **b** Fatty acid and TAG metabolism. Red (upregulated) and blue (downregulated) log_2_(fold change) values represent the expression in the 3-MA group compared to the control group. See Additional file 1: Table S1 for more details on the RNA-seq data

**Fig. 8:**
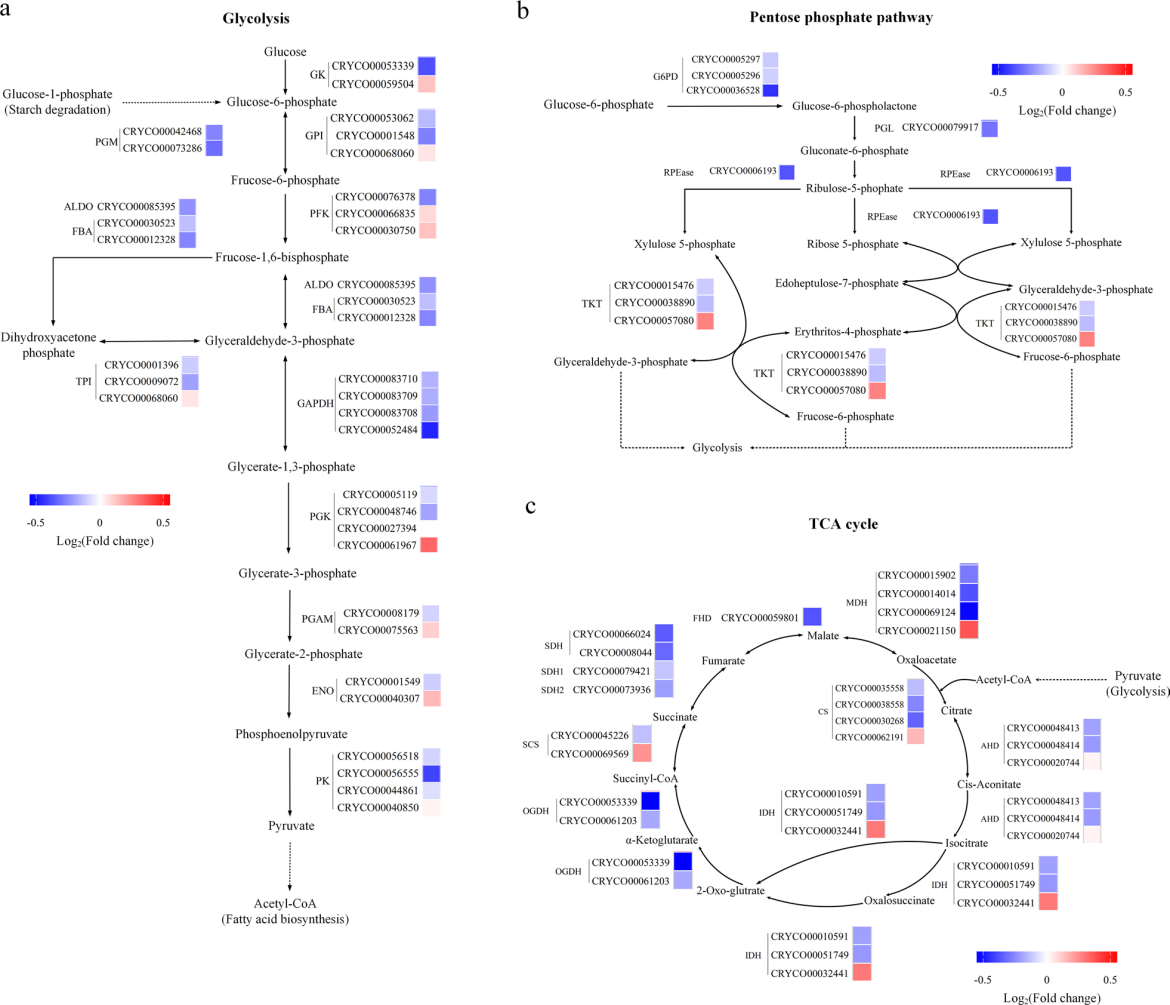
Expression of genes in carbon, ATP, NADPH metabolism in *C*. sp. SUN after 48 h. **a** Glycolysis pathway. **b** Pentose phosphate pathway. **c** TCA cycle. Red (upregulated) and blue (downregulated) log_2_(fold change) values represent the expression in the 3-MA group compared to the control group. See Additional file 1: Table S1 for more details on the RNA-seq data

**Fig. 9:**
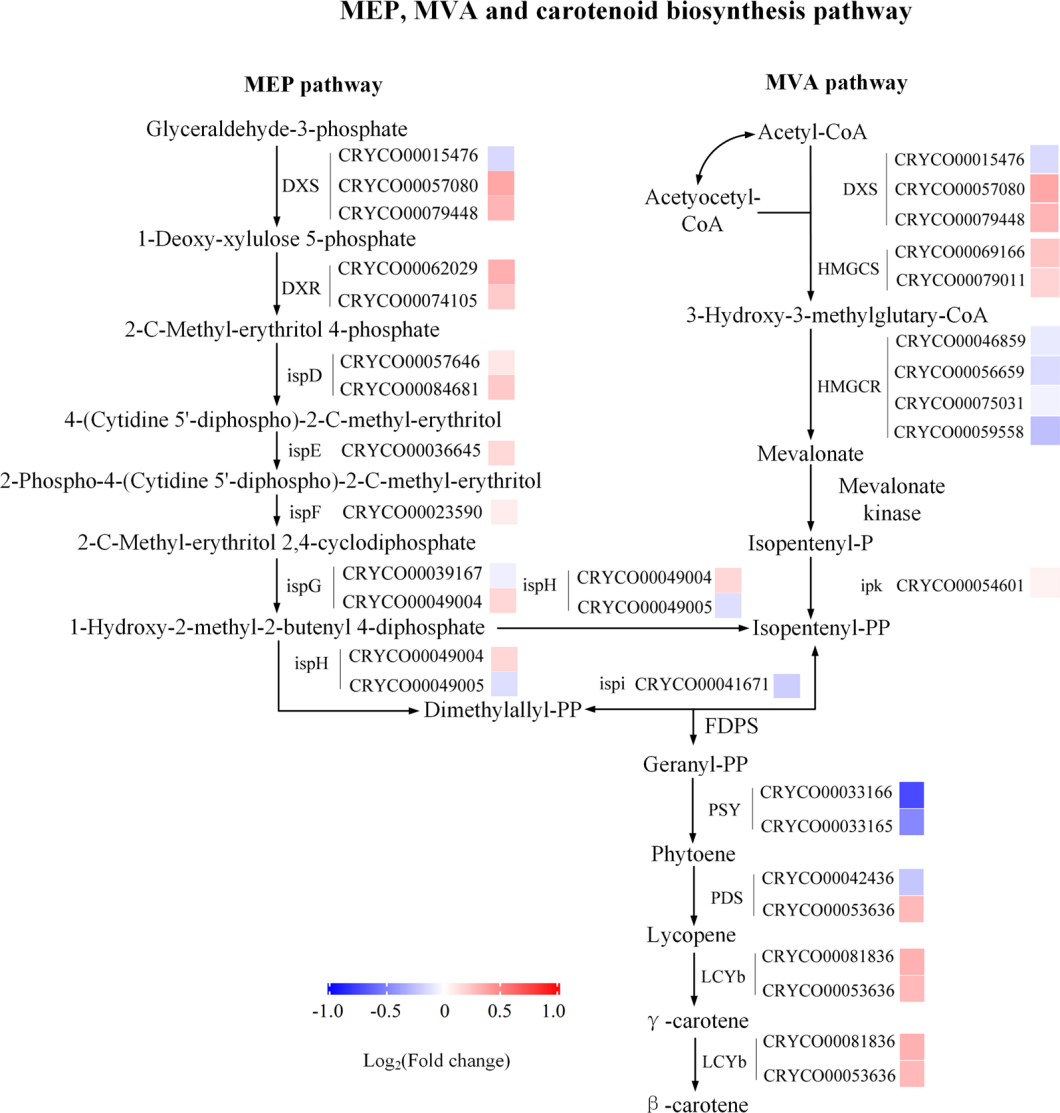
Expression of genes in carotenoid biosynthesis pathways in *C*. sp. SUN after 48 h. Red (upregulated) and blue (downregulated) log_2_(fold change) values represent the expression in the 3-MA group compared to the control group. See Additional file 1: Table S1 for more details on the RNA-seq data

Inhibition of autophagy had significant effects on autophagy progress, intracellular carbon metabolism, and energy metabolism in *C*. sp. SUN. Thus, to investigate the specific metabolic mechanisms, diagrams were constructed of the expression levels of key genes in the autophagy signaling pathway (Fig. 6), autophagy related genes (Fig. 6), starch and lipid metabolism (Fig. 7), and carbon, ATP, and NADPH metabolism pathways (Fig. 8). Besides, to study the regulatory mechanism in carotenoid pathway, the expression levels of genes in MVA, MEP, and carotenoid biosynthesis pathways (Fig. 9) were focused.

#### Autophagy signaling pathways and autophagy related genes

Autophagy refers to self-phagocytosis that is regulated by various signaling pathways, including the AMPK signaling pathway, class I PI3K/AKT signaling pathway, class III PI3K complex, and mitogen-activated protein kinase (MAPK) signaling pathway [22, 36–39]. The upregulation of class I PI3K-AKT and MAPK signaling activates TOR and reduces the initiation of autophagy [39]. Class III PI3K complex is composed of regulatory Vps15 and catalytic subunit Vps34, which are essential for the formation of autophagy vacuoles [21, 40]. 3-MA downregulated *ULK1* (also known as *ATG1*) and class III *PI3K* (Fig. 6). The AMPK signaling pathway is involved in energy sensing and signal regulation in microalgae and it is influenced by the intracellular energy status [22, 41]. A high AMP to ATP ratio can activate the AMPK pathway and inactivate TOR, and the activated AMPK phosphorylates ULK1 and promotes autophagy [41, 42]. *AMPK* was downregulated by 3-MA in *C*. sp. SUN. In addition, class I *PI3K*, *AKT*, *MAPK*, and *TOR* were all downregulated by 3-MA, which might be due to feedback regulation.

Autophagy is also regulated by autophagy related genes (*ATGs*). The ubiquitin-like conjunction mediated by ATG proteins is key to the process of autophagy. ATG12 covalently binds to ATG5 in a reaction catalyzed by ATG7 and ATG10. Thereafter, the ATG12–ATG5 protein complex non-covalently binds to an ATG16 dimer, forming a bigger protein complex [13, 43]. ATG8 (LC3 in mammals), which is another ubiquitin-like protein that is crucial in the formation of intracellular autophagic vacuoles, is cleaved by ATG4 from its carboxyl terminal to produce LC3-I, and conjugated to phosphatidyl ethanolamine (PE, a kind of PL) by ubiquitin-activating enzyme ATG7 and ubiquitin-like-conjugating enzyme ATG3 to become LCII [44]. LC3-II is recruited to the autophagosome membrane and is regarded as a typical biomarker of autophagy [44]. *ATG3*, *ATG8*, and *ATG12* were downregulated in the 3-MA group (Fig. 6), suggesting that 3-MA downregulated *ATGs*. These results are consistent with the decreased autophagic vacuoles in *C*. sp. SUN (Fig. 1).

#### Starch and lipid metabolism

Glucose is the substrate for starch synthesis in microalgae [9]. Glucose transporter (GLUT) is a membrane protein that transports glucose into the cell, which is the first step in glucose metabolism in microalgae [45]. *GLUT* was downregulated in the 3-MA group (Fig. 7a), indicating that the inhibition of autophagy downregulated glucose transport in *C*. sp. SUN. This might be the reason for the decrease in glucose consumption in the 3-MA group (Fig. 2c). In the starch biosynthesis pathway, Soluble starch synthase (SSS) and granule-bound starch synthase (GBSS), genes encoding these two essential enzymes catalyzing amylose formation, were downregulated in the 3-MA group. Most other genes involved in the starch biosynthesis pathway were also downregulated in the 3-MA group, indicating that the inhibition of autophagy downregulated the starch biosynthesis pathway in *C*. sp. SUN. These results were consistent with the lower starch level in the 3-MA group (Fig. 3a). Genes encoding isoamylase (ISA), alpha-amylase (α-AMY), and beta-amylase (β-AMY) in the starch degradation pathway were downregulated in the 3-MA group compared to the control group, suggesting that 3-MA downregulated the starch degradation pathway in *C*. sp. SUN.

Triacylglycerol (TAG) is the main storage lipid in microalgae [46, 47]. To investigate the effects of 3-MA on lipid metabolism in *C*. sp. SUN, the expression levels of genes in the fatty acid and TAG metabolism pathways were analyzed. Genes encoding acetyl-CoA carboxylase (ACCase; first rate-limiting enzyme in fatty acid synthesis), fatty acid synthase (FAS), fatty acid elongase (FAE), and fatty acid desaturase (FAD) were all downregulated in the 3-MA group compared to the control group (Fig. 7b). These results indicate that the inhibition of autophagy downregulated the fatty acid biosynthesis pathway, which is consistent with the lower TFA level in the 3-MA group (Fig. 3b). In addition, key genes in the TAG biosynthesis pathway were analyzed. Glycerol-3-phosphate O-acyltransferase (GPAT) is the rate-limiting enzyme in the TAG biosynthesis pathway, which catalyzes the formation of 1-acyl-glycerol 3-phosphate from glycerol-3-phosphate [9]. *GPAT* was downregulated in the 3-MA group. 3-MA also downregulated the genes encoding lysophosphatidic acid acyltransferase (LPAT), phosphatidate phosphatase (PAP), diacylglycerol kinase (DGK), and diacylglycerol O-acyltransferase (DGAT) in the TAG biosynthesis pathway. These results indicate that the inhibition of autophagy downregulated the TAG biosynthesis pathway in *C*. sp. SUN, which is consistent with the decrease in NLs (as a % of TFA) in the 3-MA group (Fig. 3e).

In summary, 3-MA downregulated key genes in the glucose transport system, as well as key genes involved in starch and lipid metabolism. This might be responsible for the lower glucose consumption (Fig. 2c) and the lower starch and TFA levels in the 3-MA group (Fig. 3).

#### Carbon, ATP, and NADPH metabolism

Carbon, ATP, and NADPH are needed during cell growth and fatty acid synthesis [48]. Most genes in the glycolysis pathway, pentose phosphate pathway, and TCA cycle were downregulated in the 3-MA group (Fig. 8), and only a few isoforms with low expression were upregulated, indicating that the inhibition of autophagy reduced the overall organic carbon metabolism in *C*. sp. SUN. Glyceraldehyde-3-phosphate dehydrogenase (GAPDH) and pyruvate kinase (PK) in the glycolysis pathway catalyze ATP production. *GAPDH* and *PK* were downregulated in the 3-MA group, indicating downregulation of ATP production. 6-phosphate glucose dehydrogenase (G6PD) catalyzes NADPH formation in the pentose phosphate pathway. *G6PD* was downregulated in the 3-MA group, suggesting that 3-MA decreases NADPH in *C*. sp. SUN.

ATP and NADPH are essential for fatty acid synthesis in microalgae [48, 49]. Due to the downregulation of carbon metabolism as well as the downregulation of the synthesis of ATP and NADPH, the fatty acid biosynthesis and TAG biosynthesis pathways were downregulated in the 3-MA group (Fig. 7b). Thus, this study shows that the inhibition of autophagy not only regulated the autophagy signaling pathway but also regulated the overall organic carbon and energy metabolism in *C*. sp. SUN (Figs. 6, 7  8). There may be two reasons for this phenomenon. One is that 3-MA regulates the PI3K signaling pathway and thereby affects the expression of AKT, which plays a vital role in modulating multiple cellular functions (such as cell growth, metabolism, and proliferation). The other potential reason is that by inhibiting autophagy, several ATGs were downregulated, which prevented the reuse of cytoplasmic components (such as damaged organelles) and led to the breakdown of cellular homeostasis. Therefore, regulating autophagy might be a promising strategy to promote cell growth or fatty acid accumulation in *C*. sp. SUN.

#### Carotenoid biosynthesis

Carotenoid, which is an important intracellular antioxidant, can be induced by ROS in microalgae [6, 17]. The mevalonate (MVA) and non-mevalonate (MEP) pathways are two pathways that provide precursors of carotenoids [50]. It was reported that most of green microalgae only has MEP pathway [50]. However, some microalgae species exhibit the coexistence of both the MEP pathway and the MVA pathway. For example, diatoms have been confirmed to possess both the MEP and MVA pathways through isotope tracer experiments and subsequent transcriptome sequencing [50]. In the present study, genes involved in both the MEP pathway and MVA pathway were annotated by RNA-seq. Thus, to investigate the effects of 3-MA on carotenoid biosynthesis in *C*. sp. SUN, the expression levels of genes in the MEP, MVA and carotenoid biosynthesis pathways were analyzed.

Most of the genes in the MEP pathway were significantly upregulated in the 3-MA group (Fig. 9) indicating that the inhibition of autophagy upregulated the MEP pathway. Additionally, genes encoding 1-deoxy-D-xylulose-5-phosphate synthase (DXS) and hydroxymethylglutaryl-CoA synthase (HMGCS) in the MVA pathway were significantly upregulated in the 3-MA group. However, genes encoding hydroxymethylglutaryl-CoA reductase (HMGCR) was downregulated in the 3-MA group (Fig. 4). HMGCR, which catalyzes the formation of mevalonate from 3-hydroxy-3-methylglutaryl-CoA, is a key regulatory enzyme in the MVA pathway [50]. Phytoene synthase (*PSY*), which encodes a key rate-limiting enzyme that catalyzes phytoene formation in the carotenoid biosynthesis pathway, was significantly downregulated in the 3-MA group. This is consistent with the lower total carotenoid level in the 3-MA group (Fig. 4a). Although the downregulation of *PSY* reduced carotenoid synthesis, the upregulation of *LCYb* (encoding lycopene beta cyclase, which converts γ- to β-carotene) increased the β- to γ-carotene ratio in the 3-MA group (Fig. 4b). These results indicate that *PSY* and *HMGCR* downregulation might be the key rate-limiting factors regarding carotenoid accumulation in the 3-MA group in *C*. sp. SUN.

#### Real-time quantitative PCR analysis

Nine genes (*GK, FBA, PK, IDH, OGDH, MDH, ACCase, KAS, and DGAT2*) from the glycolysis pathway, TCA cycle, and fatty acid and TAG biosynthesis pathways were selected for real-time quantitative PCR (RT-qPCR) analysis to verify the accuracy of the comparative transcriptome data. The correlation of the expression levels of the 9 genes was calculated based on Log_2_ (Fold change) values obtained from both RT-qPCR and RNA-seq. The resulting R^2^ value was 0.9191 (Additional file 2: Fig. S1), indicating a high degree of correlation and suggesting the reliability of the RNA-seq data in this study.

### Effects of rapamycin on DW, TFA and DHA content in *C*. sp. SUN

**Fig. 10:**
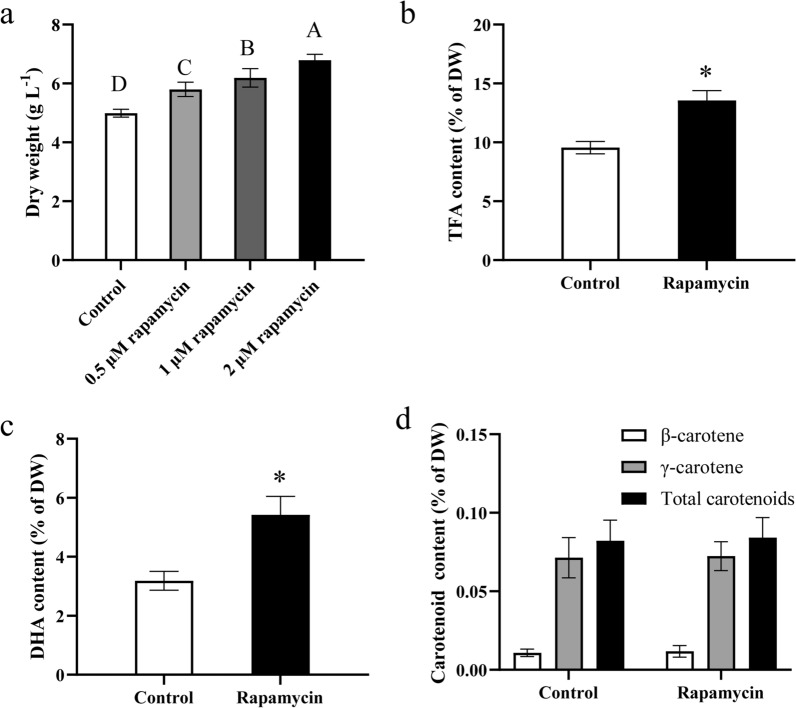
Effects of rapamycin treatment in *C*. sp. SUN after 48 h. **a** Dry weight (DW) under various concentrations (0, 0.5, 1 and 2 μM) of rapamycin treatment. **b**–**d** Effects of 2 μM rapamycin treatment on TFA content (**b**), DHA content (**c**), and carotenoid content (**d**). The letters A, B, C, and D (above the bars) in (**a**) represent the significance from high to low; * p < 0.05 vs control group in (**b**) and (**c**). Three biological replicates were conducted in each group. Data represent mean ± standard deviation

Studies have shown that TOR kinase can regulate cell growth by promoting anabolic processes (translation, ribosome biogenesis, and transcription) and antagonizing catabolic processes (autophagy and mRNA degradation) in a number of organisms [51, 52]. Rapamycin is a well-established autophagy activator known for its specific action on TOR (target of rapamycin), which can be activated by class I PI3K and AKT (downstream of class I PI3K) [26]. The results of 3-MA in this study showed that the downregulated class I PI3K might decrease the overall carbon metabolism, suggesting the class I PI3K might be a key regulating factor which affecting fatty acids and carotenoids accumulation in *C*. sp. SUN. Thus, it is reasonable to speculate that TOR also may be a key factor in regulating autophagy and further affecting fatty acids and carotenoids accumulation in *C*. sp. SUN. To investigate the effects of autophagy activation on cell growth, fatty acids and carotenoids accumulation in *C*. sp. SUN, various final concentrations of rapamycin (0, 0.5, 1 and 2 μM) in the medium were set. The autophagy activator rapamycin significantly increased the DW (Fig. 10a), indicating that rapamycin might promote cell growth in *C.* sp. SUN. The DW peaked at a rapamycin concentration of 2 μM (Fig. 10a), thus 2 μM was selected for use in subsequent experiments. The TFA and DHA levels in the 2 μM rapamycin group at 48 h was 13.56 and 5.42% of DW, which was 1.42- and 1.70-fold greater than that in the control group (Fig. 10b, c). Besides, the total carotenoid level did not significantly differ between the control and 2 μM rapamycin groups (Fig. 10d). Therefore, it can be preliminarily confirmed that the activation of autophagy increases cell growth and DHA accumulation. The results were consistent with previous studies of *Chlamydomonas* and *Cyanidioschyzon merolae*: with the inhibition of TOR and the activation of autophagy, both microalgae exhibited an accumulation of lipid bodies and NLs as well as upregulation of the key genes involved in TAG synthesis [53].

Moreover, previous studies on other microalgae have illustrated that autophagy is typically triggered in response to stressful conditions, leading to the arrest of cell growth and initiation of metabolite accumulation [10, 15]. However, the effects of autophagy initiation on cell growth and metabolite accumulation in the heterotrophic microalga *C*. sp. SUN may deviate from observations in other microalgae. The addition of the autophagy inhibitor 3-MA significantly reduced glucose absorption and utilization in *C.* sp. SUN. Concurrently, inhibiting autophagy may diminish overall carbon metabolism (including glucose metabolism and fatty acid biosynthesis) by downregulating class I PI3K (Figs. 7, 8, 9). On the contrary, the addition of rapamycin increased both cell growth and total fatty acid content (Fig. 10a, b). This effect might be attributed to rapamycin promoting autophagy, which enhances the uptake and utilization of glucose in cells and upregulates the overall organic carbon metabolism.

Overall, this study indicates the important regulatory role of autophagy in fatty acids and carotenoids accumulation in *C*. sp. SUN. An autophagy activator or inhibitor can be used to regulate fatty acids and carotenoids accumulation. This will help to inspired novel approaches for the DHA and carotenoids accumulation in *C*. sp. SUN.

### Proposed metabolic mechanisms

**Fig. 11:**
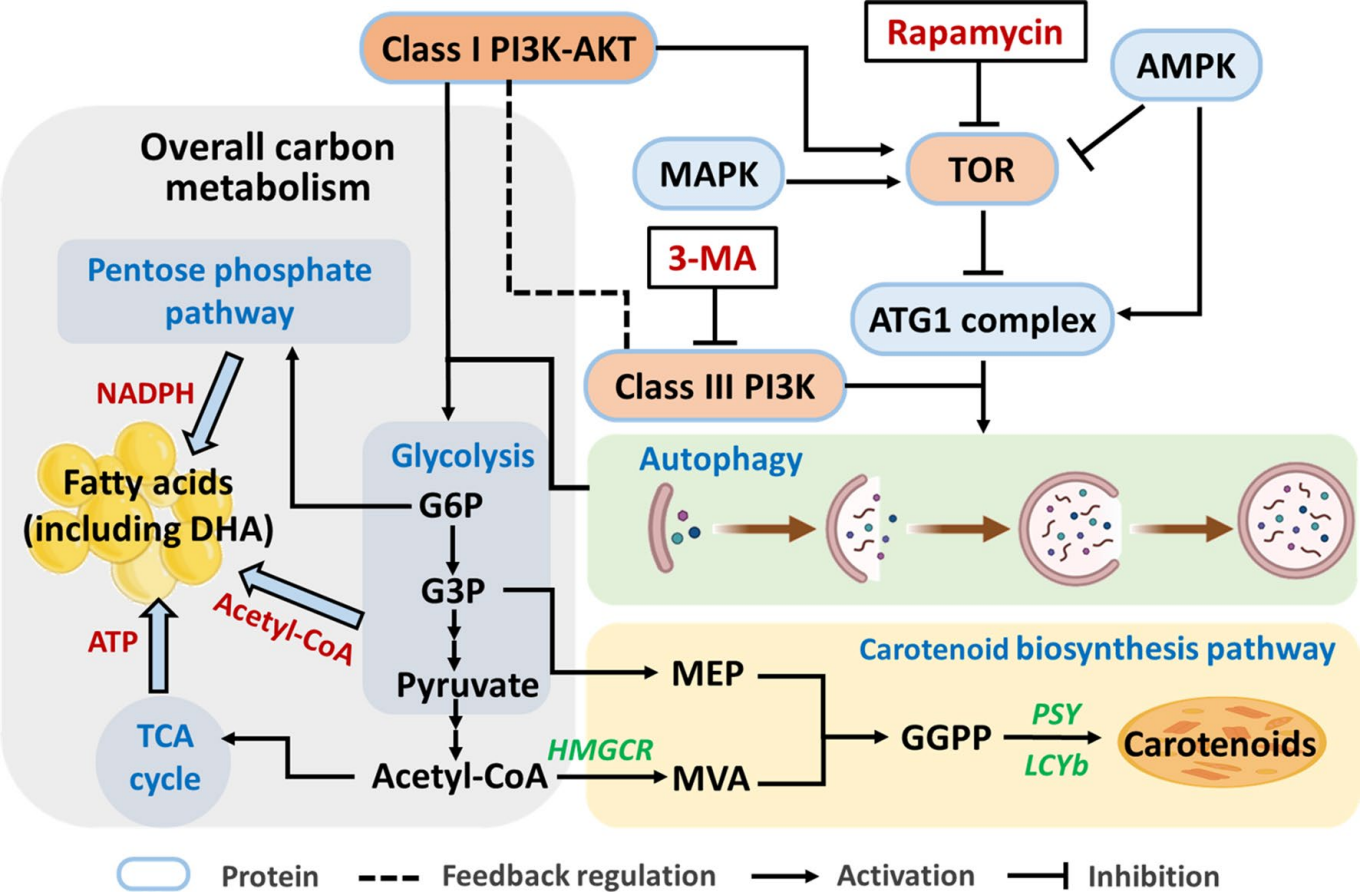
Schematic diagram of the metabolic mechanisms in *C*. sp. SUN after 3-MA treatment. *3-MA* 3-methyladenine, *PI3K* phosphoinositide 3-kinase, *AKT* protein kinase B, *TOR* target of rapamycin, *AMPK* adenosine 5‘-monophosphate (AMP)-activated protein kinase, *MAPK* mitogen-activated protein kinase, *ATG1* autophagy-related protein 1, *G6P* glucose 6‐phosphate, *G3P* glyceraldehyde-3-phosphate, *TCA* tricarboxylic acid, *NADPH* nicotinamide adenine dinucleotide phosphate, *ATP* adenosine triphosphate, *DHA* docosahexaenoic acid, *MEP* 2-C-methyl-erythritol 4-phosphate, *MVA* mevalonate, *GGPP* geranylgeranyl diphosphate, *HMGCR* hydroxymethylglutaryl-CoA reductase, *LCYb* lycopene beta cyclase, *PSY* phytoene synthase

A schematic diagram of regulating autophagy to increase DHA and carotenoids accumulation is concluded and presented in Fig. 11. Autophagy inhibitor 3-MA specifically inhibits class III PI3K, thereby suppressing autophagy progress. The inactivation of class III PI3K may feedback on class I PI3K, which downregulates the overall carbon metabolism. Thus, the reduction of acetyl-CoA, NADPH and ATP supply (form glycolysis pathway, pentose phosphate pathway and TCA cycle, respectively) would lead to the decrease of TFA (including DHA) accumulation. Besides, inhibition of autophagy lead to the downregulation of *HMGCR* and *PSY*, underlies the decrease in total carotenoids content. From perspective of activation of autophagy, rapamycin can activate autophagy by regulating TOR (target of rapamycin). Then the overall carbon metabolism may upregulate and provide more acetyl-CoA, NADPH and ATP for DHA biosynthesis. Therefore, the DHA content was increased with the rapamycin treatment. In summary, this schematic diagram provides a possible action path for the mechanisms of increasing DHA and carotenoids accumulation by regulating autophagy.

## Conclusions

In this study, the inhibition of autophagy significantly downregulated carbon metabolism, which likely contributed to the decrease of TFA content and DHA content by the decrease of the acetyl-CoA, NADPH and ATP supply. The class I PI3K-AKT signaling pathway may be crucial for the regulation of carbon metabolism. Besides, 3-MA significantly increased ROS but downregulated *PSY* and *HMGCR*, underlies the decrease in total carotenoids content. Thus, the autophagy activator rapamycin was employed, and significantly enhanced the cell growth, TFA and DHA accumulation. This study provides insights for the manipulation of DHA metabolism by regulating autophagy.

## Methods

### Strain and culture conditions

The *Crypthecodinium* sp. SUN strain preserved in our laboratory was isolated from Longhai National Mangrove Natural Reserve [6]. By + medium (20 g L^−1^ sea salt, 20 g L^−1^ glucose, 1 g L^−1^ yeast extract, and 1 g L^−1^ tryptone) was used to cultivate this strain [5]. The original seed culture was preserved at 16 °C in the dark. To obtain the active seed culture, the original seed culture was inoculated in a 250 mL Erlenmeyer flask containing 50 mL fresh By + medium. The cells were then cultured at 25 °C with orbital shaking (150 rpm) in the dark. After 96 h of cultivation, the seed culture was inoculated into fresh medium based on a 10% (v/v) inoculation dose and cultured in the light (100 μmol photons m^−2^ s^−1^). To inhibit autophagy, 3-MA was added during the cultivation of *C*. sp. SUN, with a final concentration of 3 mM. Samples were taken to measure key indexes at 0, 24, 48, 72 and 96 h, respectively.

### Observation by transmission electron microscopy

Samples were prepared according to the method described by Perez-Perez et al. and Zhang et al. [19, 54]. The collected cells were fixed with 2.5% glutaraldehyde for 4 h, washed 3 times with phosphate-buffered saline, fixed with 1% osmic acid at 4 °C for 2 h, progressively dehydrated with ethanol from 30 to 100%, treated with 100% propylene oxide, and embedded using 812 resin (Spi-Chem, New York, America). After polymerization at 60 °C, the embedded blocks were sliced using an ultra-thin microtome (Leica UC7, Leica, Austria). Finally, the sections were stained and observed by TEM (HT7700, HITACHI, Japan).

### Measurement of cell number and dry weight

To determine the cell number in the control and 3-MA groups, 1 mL culture of each sample (i.e., for each time point in each group) was collected into a new tube. About 10 μL culture was dropped onto a hemocytometer and the cells were counted using an optical microscope (ECLIPSE Ei R, Nikon, Shanghai, China).

To quantify dry weight (DW), 5 mL culture was subjected to centrifugation. The pellet was then washed twice with distilled water to remove the culture medium, subjected to suction filtration, loaded onto pre-weighed filter papers (No. 1440–070, Whatman, UK), and dried at 80 °C for 4 h in a vacuum drying oven (DZF-6050, Yiheng, Shanghai, China). The difference in mass before (m_0_) and after (m_1_) drying was used to calculate the DW using the following formula:

$${\text{DW}}\left( {{\text{g L}}^{{ - {1}}} } \right) = \, [{\text{m}}_{{1}} \left( {\text{g}} \right) - {\text{m}}_{0} \left( {\text{g}} \right)]/ \, ({5} \times {1}0^{{ - {3}}} {\text{L}})$$

### Measurement of residual glucose concentration

1 mL culture was subjected to centrifugation (8000 rpm for 5 min) and the supernatant was used to measure the glucose consumption. Briefly, 200 μL supernatant was added to a tube, followed by 1 mL 3,5-dinitrosalicylic acid (DNS), and incubated at 100 ℃ [9, 55]. The absorbance at 570 nm was measured using a microplate reader (SpectraMax iD3, Molecular Devices, Shanghai, China). The residual glucose concentration was calculated using a glucose standard curve.

### Measurement of intracellular reactive oxygen species

Intracellular ROS in *C.* sp. SUN was quantified using the method described by Zhang et al. using a Reactive Oxygen Species Assay Kit (No. S0033M, Beyotime, China) [17]. 1 mL culture was subjected to centrifugation (8000 rpm for 5 min) and the precipitated cells were then mixed with 200 μL By + medium containing 10 μM 2′, 7′-dichlorofluorescin diacetate (DCFH-DA; fluorescent probe), incubated for 20 min in the dark, washed 3 times with fresh By + medium, and resuspended in the original culture medium. The fluorescence intensity was measured on a microplate reader (SpectraMax iD3, Molecular Devices, Shanghai, China) at 485 nm (excitation wavelength) and 520 nm (emission wavelength).

### Measurement of starch

About 60 mg lyophilized algae cells was weighed and thoroughly ground in a mortar to disrupt the cell wall [17]. The cell debris was washed 3 times using Tris/HCl buffer (20 mM, pH 6.9) and then centrifuged at 12,000 rpm at 4 °C for 10 min to collect the sediment. To remove pre-existing glucose and maltose, 5 mL ethanol solution (80%) was added and incubated at 85 °C for 5 min before immediately cooling the mixture. The mixture was then centrifuged at 12,000 rpm at 4 °C for 10 min. To digest the resistant starch, 2 mL dimethyl sulfoxide (DMSO) was added to the pellet and the mixture was incubated in a boiling water bath for 5 min. To resolve the starch, 4 mL Tris/HCl buffer and 20 μL thermally stabilized α-amylase were added and incubated again in a boiling water bath for 5 min. After cooling on ice, glucoamylase was added and incubated in a shaking bath at 60 °C for 15 min. The supernatant was collected after centrifugation at 8000 rpm at 4 °C for 5 min to measure the concentration of glucose. Finally, the starch level was calculated based on the glucose concentration [55].

### Measurement of fatty acids

About 20 mg lyophilized algal cells was weighed and the methyl esterification of fatty acid was performed in a in a heat-resistant glass tube with a lid. 1 mL toluene, 2 mL 1% sulfuric acid dissolved in methanol (v/v), and 0.5 mL heptadecanoic acid (C17:0) were added [17]. The mixture was heat treated at 85 °C for 3 h. Next, the fatty acid methyl esters (FAMEs) were extracted and analyzed using a gas chromatograph (GC) (7890A, Agilent, USA) with a DB-23 capillary column (30 m × 0.25 mm × 0.25 µm, Agilent, USA) [9].

### Total lipid extraction and analysis

About 60 mg lyophilized algal cells was weighed and thoroughly ground in a mortar. A mixture of chloroform and methyl alcohol (2:1) was used 3 times to extract the lipids. To prevent lipid oxidation, 0.01% butylated hydroxytoluene (BHT) was added. The extracts were combined and washed with 0.75% NaCl solution to remove the soluble protein and remaining cell debris. After centrifuging at 8000 rpm at 4 °C for 5 min, the chloroform layer was collected for the following analysis.

The extracted total lipids were separated into three subclasses: neutral lipids (NLs), glycolipids (GLs), and phospholipids (PLs). For solid-phase extraction, a special column, namely 500 mg Sep-PakTM silica cartridge (WAT43400, Sep-Pak Vac 6cc, Waters, USA), was used. Briefly, 10 mL chloroform was added to activate the column, the extracted total lipids were added to the column, and then chloroform, acetone, and methanol were used to elute the NLs, GLs, and PLs, respectively [5]. Each eluent was dried with nitrogen gas and quantified by GC (7890A, Agilent, USA) after methyl esterification.

### Measurement of protein

About 2 mg lyophilized algal cells was weighed to measure the protein level according to the bicinchoninic acid (BCA) method [9]. After alkali treatment (3% KOH solution) for 10 min, the broken cells were washed 3 times to obtain the crude protein extract, which underwent centrifugation at 8000 rpm for 5 min. The supernatant was used with a BCA protein measurement kit (No. P0012, Beyotime, China) to measure the protein level.

### Carotenoid extraction and quantification

About 30 mg lyophilized algal cells was weighed, ground, and underwent extraction twice with acetone until the algal residue became colorless. After drying with nitrogen, the extracted carotenoids were dissolved in 1 mL chromatographic-grade acetone. High-performance liquid chromatography (HPLC) was used to quantify the carotenoid level. The chromatographic column was a C18 column. The volume of each injection was 10 μL. There were four mobile phases: (A) pure ethyl acetate; (B) acetonitrile: methanol: ultrapure water (84: 2: 14); (C) methanol: ultrapure water (10:90); and (D) pure methanol [6].

### Comparative transcriptome analysis

To quickly collect the fresh algal cells, 20 mL culture were centrifuged at 12,000 rpm at 25 °C. Then the cells were fully ground under the protection of liquid nitrogen. Total RNA of the cells was extracted using RNAiso plus reagent (No. 9108/9109, Takara, Beijing, China) and checked for quality. To ensure the accuracy of the data, high-quality RNA samples were used to construct the sequencing library. To obtain cDNA, a Super Script double-stranded cDNA synthesis kit (No. 11917–020, Invitrogen, Waltham, USA) was used. An Illumina sequencer (NovaSeq 6000, Shanghai, China) was used to obtain the final RNA-seq library for the following analysis. Transcripts per million reads (TPM) was used to calculate the expression level of each gene or transcript. Then, the expressed genes and transcripts were analyzed by functional database annotation using Non-Redundant Protein Sequence Database (NR), Gene Ontology (GO), Clusters of Orthologous Groups (COG), Kyoto Encyclopedia of Genes and Genomes (KEGG), Swiss-Port Protein Sequence Database (Swiss-Port), and the protein family database (Pfam)). The log_2_(fold change) values represent the expression in the 3-MA group compared to the control group. Genes with adjusted *p* < 0.05 and |log_2_(fold change)|≥ 1 between the 3-MA vs control group were designated differentially expressed genes. All differentially expressed genes were included in a gene set for further analysis. Lastly, functional-enrichment analyses were performed, including Kyoto Encyclopedia of Genes and Genomes (KEGG) analysis.

### Real-time quantitative PCR for transcriptome data validation

To verify the reliability of the transcriptome data, nine genes were selected to conduct real-time quantitative PCR (RT-qPCR) using a CFXTM Real-Time System (Bio-Rad, Hercules, USA). The primer sequences (Additional file 2: Table S1) were designed using Primer Premier 5 software. The total volume of the system was 20 μL. The expression levels of the nine genes were evaluated by the 2^−∆∆CT^ method, and the actin (*ACT*) gene of *C*. sp. SUN was used as the control [56]. The correlation of the expression levels of the 9 genes (*GK*, *FBA*, *PK*, *IDH*, *OGDH*, *MDH*, *ACCase*, *KAS* and *DGAT2*) based on Log_2_ (Fold change) in RT-qPCR and RNA-seq were analyzed.

### Statistical analysis

All experiments involved three biological replicates. The data for each time point in the figures and tables are presented as mean ± standard deviation. The significance of the results was calculated by one-way analysis of variance (ANOVA) using IBM SPSS Statistics 26.0. All figures were constructed using GraphPad Prism 9.0.0.

### Supplementary Information

**Additional file 1: Table S1.** The RNA-seq data of the genes involved in the selected biological pathways. After KEGG enrichment analysis of the significant differential genes between 3-MA and control groups, several pathways including autophagy signaling pathway, starch and lipid metabolism, glycolysis, pentose phosphate pathway, TCA cycle, MEP, MVA, and carotenoid biosynthesis pathways were focused. Details about genes information and expression levels were listed in the table.

**Additional file 2: Fig S1.** The correlation of the expression levels of the 9 genes based on Log_2_ (Fold change) in both RT-qPCR and RNA-seq in C. sp. SUN after 48 h. To verify the accuracy of the comparative transcriptome data, the correlation of the expression levels of the 9 genes (GK, FBA, PK, IDH, OGDH, MDH, ACCase, KAS and DGAT2) was calculated based on Log_2_ (Fold change) values obtained from both RT-qPCR and RNA-seq. **Table S1.** RT-qPCR primers in *C*. sp. SUN. For RT-qPCR analysis, 9 genes including *GK*, *FBA*, *PK*, *IDH*, *OGDH*, *MDH*, *ACCase*, *KAS* and *DGAT2* were selected. The Table S2 showed the primers of these 9 genes in this study.

## Data Availability

All data supporting the findings of this study are available within the paper and within its supplementary materials published online.

## Abbreviations

*C*. sp. SUN: *Crypthecodinium* sp. SUN
3-MA: 3-Methyladenine
DHA (C22:6): Docosahexaenoic acid
TFA: Total fatty acid
TAG: Triacylglycerol
TEM: Transmission electron microscopy
DW: Dry weight
ROS: Reactive oxygen species
C14:0: Myristic acid
C16:0: Palmitic acid
C17:0: Heptadecanoic acid
C18:0: Stearic acid
C18:1: Oleic acid
SFAs: Saturated fatty acids
MUFAs: Monounsaturated fatty acids
PUFAs: Polyunsaturated fatty acids
NLs: Neutral lipids
GLs: Glycolipids
PLs: Phospholipids
TOR: Target of rapamycin
AMPK: Adenosine 5‘-monophosphate (AMP)-activated protein kinase
MAPK: Mitogen-activated protein kinase
TCA: Tricarboxylic acid
MVA: Mevalonate
MEP: Non-mevalonate
NADPH: Nicotinamide adenine dinucleotide phosphate
ATP: Adenosine triphosphate
AMP: Adenosine monophosphate
Vps15: Vacuolar protein sorting 15
Vps34: Vacuolar protein sorting 34
PE: Phosphatidyl ethanolamine
GLUT: Glucose transporter
SSS: Starch synthase
GBSS: Granule-bound starch synthase
ISA: Isoamylase
α-AMY: Alpha-amylase
β-AMY: Beta-amylase
ACCase: Acetyl-CoA carboxylase
FAS: Fatty acid synthase
FAE: Fatty acid elongase
FAD: Fatty acid desaturase
GPAT: Glycerol-3-phosphate O-acyltransferase
LPAT: Lysophosphatidic acid acyltransferase
PAP: Phosphatidate phosphatase
DGK: Diacylglycerol kinase
DGAT: Diacylglycerol O-acyltransferase
GAPDH: Glyceraldehyde-3-phosphate dehydrogenase
PK: Pyruvate kinase
G6PD: 6-Phosphate glucose dehydrogenase
DXS: 1-Deoxy-D-xylulose-5-phosphate synthase
HMGCS: Hydroxymethylglutaryl-CoA synthase
GK: Glucokinase
FBA: Fructose-bisphosphate aldolase
PSY: Phytoene synthase
HMGCR: Hydroxymethylglutaryl-CoA reductase
LCYb: Lycopene beta cyclase
GGPP: Geranylgeranyl pyrophosphate
ATG: Autophagy related genes
ULK1: Unc-51-like kinase 1
PBS: Phosphate buffer solution
DNS: 3,5-Dinitrosalicylic acid
DCFH-DA: 2′, 7′-Dichlorofluorescin diacetate
Tris/HCl: TRIS hydrochloride
DMSO: Dimethyl sulfoxide
FAMEs: Fatty acid methyl esters
GC: Gas chromatograph
BHT: Butylated hydroxytoluene
NaCl: Sodium chloride
BCA: Bicinchoninic acid
HPLC: High performance liquid chromatography
RNA: Ribonucleic acid
cDNA: Complementary deoxyribonucleic acid
RNA-seq: RNA sequencing
TPM: Transcripts per million reads
RT-qPCR: Real-time quantitative polymerase chain reaction
ACT: Actin
SD: Standard deviation
ANOVA: One-way analysis of variance

## References

[1] Johnson X, Alric J (2013). Central carbon metabolism and electron rransport in *Chlamydomonas reinhardtii*: metabolic constraints for carbon partitioning between oil and starch. Eukaryot Cell.

[2] Zhuang LL, Yu D, Zhang J, Liu FF, Wu YH, Zhang TY, Dao GH, Hu HY (2018). The characteristics and influencing factors of the attached microalgae cultivation: a review. Renew Sust Energ Rev.

[3] Kitson AP, Metherel AH, Chen CT, Domenichiello AF, Trepanier MO, Berger A, Bazinet RP (2016). Effect of dietary docosahexaenoic acid (DHA) in phospholipids or triglycerides on brain DHA uptake and accretion. J Nutr Biochem.

[4] Neijat M, Ojekudo O, House JD (2016). Effect of flaxseed oil and microalgae DHA on the production performance, fatty acids and total lipids of egg yolk and plasma in laying hens. Prostag Leukotr Ess.

[5] Sun D, Zhang Z, Mao X, Wu T, Jiang Y, Liu J, Chen F (2017). Light enhanced the accumulation of total fatty acids (TFA) and docosahexaenoic acid (DHA) in a newly isolated heterotrophic microalga *Crypthecodinium* sp. SUN Bioresour Technol.

[6] Sun D, Zhang Z, Zhang Y, Cheng KW, Chen F (2019). Light induces carotenoids accumulation in a heterotrophic docosahexaenoic acid producing microalga, *Crypthecodinium sp*. SUN. Bioresour Technol.

[7] Krishnan A, Kumaraswamy GK, Vinyard DJ, Gu HY, Ananyev G, Posewitz MC, Dismukes GC (2015). Metabolic and photosynthetic consequences of blocking starch biosynthesis in the green alga *Chlamydomonas reinhardtii* sta6 mutant. Plant J.

[8] Vonlanthen S, Dauvillee D, Purton S (2015). Evaluation of novel starch-deficient mutants of *Chlorella sorokiniana* for hyper-accumulation of lipids. Algal Res.

[9] Li YM, Tian WN, Fu ZX, Ye WQ, Zhang XW, Zhang Z, Sun DZ (2022). Mechanisms of sodium-acetate-induced DHA accumulation in a DHA-producing microalga, *Crypthecodinium* sp. SUN. Mar Drugs.

[10] Xi Y, Kong F, Chi Z (2020). ROS induce beta-carotene biosynthesis caused by changes of photosynthesis efficiency and energy metabolism in *Dunaliella salina* under stress conditions. Front Bioeng Biotechnol.

[11] Zhang LJ, Pei HY, Chen SQ, Jiang LQ, Hou QJ, Yang ZG, Yu Z (2018). Salinity-induced cellular cross-talk in carbon partitioning reveals starch-to-lipid biosynthesis switching in low-starch freshwater algae. Bioresour Technol.

[12] Tran QG, Yoon HR, Cho K, Lee SJ, Crespo JL, Ramanan R, Kim HS (2019). Dynamic interactions between autophagosomes and lipid droplets in *Chlamydomonas reinhardtii*. Cells.

[13] Kajikawa M, Yamauchi M, Shinkawa H, Tanaka M, Hatano K, Nishimura Y, Kato M, Fukuzawa H (2019). Isolation and characterization of *Chlamydomonas* autophagy-related mutants in nutrient-deficient conditions. Plant Cell Physiol.

[14] Pugkaew W, Meetam M, Ponpuak M, Yokthongwattana K, Pokethitiyook P (2017). Role of autophagy in triacylglycerol biosynthesis in *Chlamydomonas reinhardtii* revealed by chemical inducer and inhibitors. J Appl Phycol.

[15] Liao Q, Chang HX, Fu Q, Huang Y, Xia A, Zhu X, Zhong N (2018). Physiological-phased kinetic characteristics of microalgae *Chlorella vulgaris* growth and lipid synthesis considering synergistic effects of light, carbon and nutrients. Bioresour Technol.

[16] Couso I, Perez-Perez ME, Martinez-Force E, Kim HS, He Y, Umen JG, Crespo JL (2018). Autophagic flux is required for the synthesis of triacylglycerols and ribosomal protein turnover in *Chlamydomonas*. J Exp Bot.

[17] Zhang Z, Sun D, Cheng KW, Chen F (2018). Inhibition of autophagy modulates astaxanthin and total fatty acid biosynthesis in *Chlorella zofingiensis* under nitrogen starvation. Bioresour Technol.

[18] Wang X, Song Y, Liu B, Hang W, Li R, Cui H, Li R, Jia X (2020). Enhancement of astaxanthin biosynthesis in *Haematococcus pluvialis* via inhibition of autophagy by 3-methyladenine under high light. Algal Res.

[19] Perez-Perez ME, Couso I, Heredia-Martinez LG, Crespo JL (2017). Monitoring autophagy in the model green microalga *Chlamydomonas reinhardtii*. Cells.

[20] Thompson AR, Doelling JH, Suttangkakul A, Vierstra RD (2005). Autophagic nutrient recycling in *Arabidopsis* directed by the ATG8 and ATG12 conjugation pathways. Plant Physiol.

[21] Dibble CC, Barritt SA, Perry GE, Lien EC, Geck RC, DuBois-Coyne SE, Bartee D, Zengeya TT, Cohen EB, Yuan M (2022). PI3K drives the de novo synthesis of coenzyme A from vitamin B5. Nature.

[22] Kma L, Baruah TJ (2022). The interplay of ROS and the PI3K/Akt pathway in autophagy regulation. Biotechnol Appl Biochem.

[23] Shang L, Wang X (2011). AMPK and mTOR coordinate the regulation of Ulk1 and mammalian autophagy initiation. Autophagy.

[24] Perez-Perez ME, Florencio FJ, Crespo JL (2010). Inhibition of target of rapamycin signaling and stress activate autophagy in *Chlamydomonas reinhardtii*. Plant Physiol.

[25] Dibble CC, Cantley LC (2015). Regulation of mTORC1 by PI3K signaling. Trends Cell Biol.

[26] Xu F, Na L, Li Y, Chen L (2020). Roles of the PI3K/AKT/mTOR signalling pathways in neurodegenerative diseases and tumours. Cell Biosci.

[27] Kim J, Kundu M, Viollet B, Guan KL (2011). AMPK and mTOR regulate autophagy through direct phosphorylation of Ulk1. Nat Cell Biol.

[28] Russell RC, Yuan HX, Guan KL (2014). Autophagy regulation by nutrient signaling. Cell Res.

[29] Jiang Q, Zhao L, Dai J, Wu Q (2012). Analysis of autophagy genes in microalgae: *Chlorella* as a potential model to study mechanism of autophagy. PLoS ONE.

[30] Kokabi K, Gorelova O, Zorin B, Didi-Cohen S, Itkin M, Malitsky S, Solovchenko A, Boussiba S, Khozin-Goldberg I (2020). Lipidome remodeling and autophagic respose in the arachidonic-acid-rich microalga *Lobosphaera incisa* under nitrogen and phosphorous deprivation. Front Plant Sci.

[31] Yoshitake Y, Ohta H, Shimojima M (2019). Autophagy-mediated regulation of lipid metabolism and its impact on the growth in algae and seed plants. Front Plant Sci.

[32] Rezayian M, Niknam V, Ebrahimzadeh H (2019). Oxidative damage and antioxidative system in algae. Toxicol Rep.

[33] Jothibasu K, Dhar DW, Rakesh S (2021). Recent developments in microalgal genome editing for enhancing lipid accumulation and biofuel recovery. Biomass Bioenerg.

[34] Wang L, Huang X, Lim DJ, Laserna AKC, Li SFY (2019). Uptake and toxic effects of triphenyl phosphate on freshwater microalgae *Chlorella vulgaris* and *Scenedesmus obliquus*: insights from untargeted metabolomics. Sci Total Environ.

[35] Ding W, Li QQ, Han BY, Zhao YT, Geng SX, Ning DL, Ma T, Yu XY (2019). Comparative physiological and metabolomic analyses of the hyper-accumulation of astaxanthin and lipids in *Haematococcus pluvialis* upon treatment with butylated hydroxyanisole. Bioresour Technol.

[36] Kajikawa M, Fukuzawa H (2020). Algal autophagy is necessary for the regulation of carbon metabolism under nutrient deficiency. Front Plant Sci.

[37] McLoughlin F, Marshall RS, Ding X, Chatt EC, Kirkpatrick LD, Augustine RC, Li F, Otegui MS, Vierstra RD (2020). Autophagy plays prominent roles in amino acid, nucleotide, and carbohydrate metabolism during fixed-carbon starvation in maize. Plant Cell.

[38] Desideri E, Vegliante R, Cardaci S, Nepravishta R, Paci M, Ciriolo MR (2014). MAPK14/p38alpha-dependent modulation of glucose metabolism affects ROS levels and autophagy during starvation. Autophagy.

[39] Schultze SM, Hemmings BA, Niessen M, Tschopp O (2012). PI3K/AKT, MAPK and AMPK signalling: protein kinases in glucose homeostasis. Expert Rev Mol Med.

[40] Hopkins BD, Goncalves MD, Cantley LC (2020). Insulin-PI3K signalling: an evolutionarily insulated metabolic driver of cancer. Nat Rev Endocrinol.

[41] van Janse Rensburg HC, Van den Ende W, Signorelli S (2019). Autophagy in plants: both a puppet and a puppet master of sugars. Front Plant Sci.

[42] Herzig S, Shaw RJ (2018). AMPK: guardian of metabolism and mitochondrial homeostasis. Nat Rev Mol Cell Biol.

[43] Fischer S, Rijal R, Frommolt P, Wagle P, Konertz R, Faix J, Messling S, Eichinger L (2019). Functional characterization of ubiquitin-like core autophagy protein ATG12 in *dictyostelium discoideum*. Cells.

[44] Nakatogawa H, Oh-oka K, Ohsumi Y (2008). Lipidation of Atg8: how is substrate specificity determined without a canonical E3 enzyme?. Autophagy.

[45] Huang G, Zhao D, Lan C, Wu B, Li X, Lou S, Zheng Y, Huang Y, Hu Z, Jia B (2022). Glucose-assisted trophic conversion of *Chlamydomonas reinhardtii* by expression of glucose transporter GLUT1. Algal Res.

[46] Ma XM, Mi YW, Zhao C, Wei Q (2022). A comprehensive review on carbon source effect of microalgae lipid accumulation for biofuel production. Sci Total Environ.

[47] Zhao TT, Liu MX, Zhao TT, Chen AL, Zhang L, Liu H, Ding K, Xiao PY (2021). Enhancement of lipid productivity in *Chlorella pyrenoidosa* by collecting cells at the maximum cell number in a two-stage culture strategy. Algal Res.

[48] Burlacot A, Peltier G, Li-Beisson Y (2019). Subcellular energetics and carbon storage in *Chlamydomonas*. Cells.

[49] Osada K, Maeda Y, Yoshino T, Nojima D, Bowler C, Tanaka T (2017). Enhanced NADPH production in the pentose phosphate pathway accelerates lipid accumulation in the oleaginous diatom *Fistulifera solaris*. Algal Res.

[50] Huang PW, Wang LR, Geng SS, Ye C, Sun XM, Huang H (2021). Strategies for enhancing terpenoids accumulation in microalgae. Appl Microbiol Biotechnol.

[51] Perez-Perez ME, Couso I, Crespo JL (2017). The TOR signaling network in the model unicellular green alga *Chlamydomonas reinhardtii*. Biomolecules.

[52] Gonzalez A, Hall MN (2017). Nutrient sensing and TOR signaling in yeast and mammals. Embo J.

[53] Imamura S, Kawase Y, Kobayashi I, Sone T, Era A, Miyagishima SY, Shimojima M, Ohta H, Tanaka K (2015). Target of rapamycin (TOR) plays a critical role in triacylglycerol accumulation in microalgae. Plant Mol Biol.

[54] Zhang Z, Sun D, Chen F (2020). Comparative transcriptome analysis revealing the mechanisms underlying light-induced total fatty acid and carotenoid accumulation in *Crypthecodinium* sp. SUN. Algal Res.

[55] Zhang Z, Sun D, Zhang Y, Chen F (2019). Glucose triggers cell structure changes and regulates astaxanthin biosynthesis in *Chromochloris zofingiensis*. Algal Res.

[56] Sirikhachornkit A, Suttangkakul A, Vuttipongchaikij S, Juntawong P (2018). De novo transcriptome analysis and gene expression profiling of an oleaginous microalga *Scenedesmus acutus* TISTR8540 during nitrogen deprivation-induced lipid accumulation. Sci Rep-Uk.

