# Supplementary material for: Regulatory mechanisms of autophagy on DHA and carotenoid accumulation in Crypthecodinium sp. SUN
Source: Biotechnol Biofuels Bioprod. 2024 Apr 2;17:50. doi: 10.1186/s13068-024-02493-6 (PMC10985998; doi:10.1186/s13068-024-02493-6)
Supplement: Supplementary file 2 — Additional file 2: Fig S1. The correlation of the expression levels of the 9 genes based on Log2 (Fold change) in both RT-qPCR and RNA-seq in C. sp. SUN after 48 h. To verify the accuracy of the comparative transcriptome data, the correlation of the expression levels of the 9 genes (GK, FBA, PK, IDH, OGDH, MDH, ACCase, KAS and DGAT2) was calculated based on Log2 (Fold change) values obtained from both RT-qPCR and RNA-seq. Table S1. RT-qPCR primers in C. sp. SUN. For RT-qPCR analysis, 9 genes including GK, FBA, PK, IDH, OGDH, MDH, ACCase, KAS and DGAT2 were selected. The Table S2 showed the primers of these 9 genes in this study. [file 13068_2024_2493_MOESM2_ESM.doc]

**
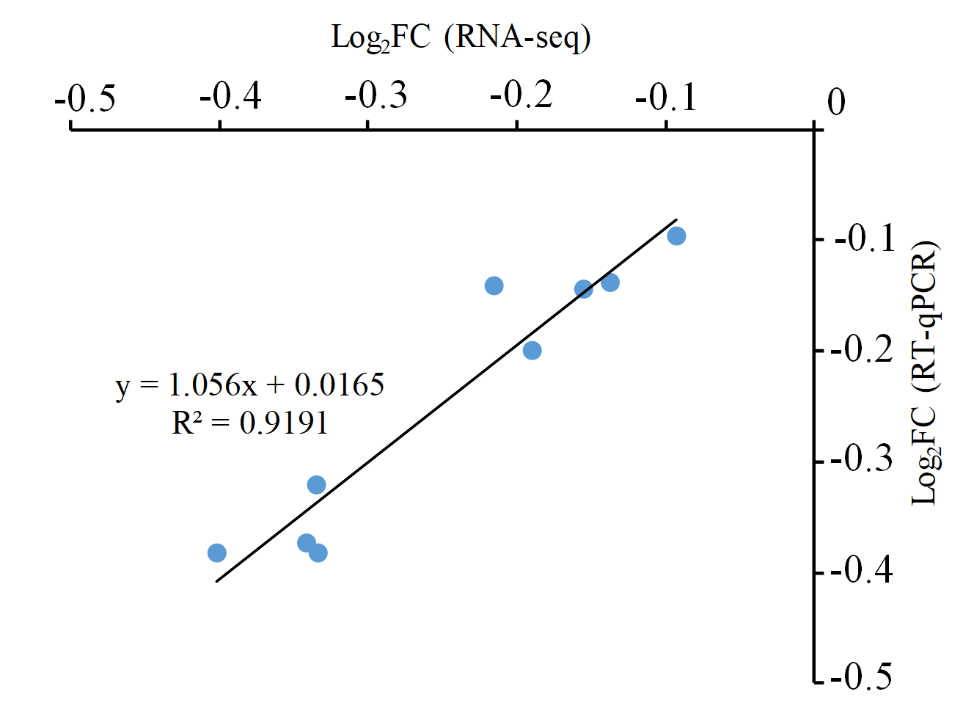
**

**Figure S1** The correlation of the expression levels of the 9 genes based on Log_2_ (Fold change) in both RT-qPCR and RNA-seq in *C*. sp. SUN after 48 h.

**Table S1** RT-qPCR primers in *C*. sp. SUN

| Gene name | Forward primer (5’-3’) | Reverse primer (5’-3’) |
| --- | --- | --- |
| GK | TGTGATGTATGCCCAAGCGATGA | AAGCCAGTGTTCAGGATGGGAGTG |
| FBA | TGATGTATGCCCAAGCGATGACG | GCATACATCACAGGGCGAGGGAC |
| PK | TGGACTTCGTCGCTGCCTCTTT | AGAGGCAGCGACGAAGTCCACA |
| IDH | TTCTTCGCCCGATGCTTGGAGG | AGGGCACGATGTTCGCCTCCAA |
| OGDH | GAAACGATGCCTTGGGTATGGA | GACGGGCACAACGAGACCTTT |
| MDH | ACAAGTTCGTCGGCACTATCACA | TGTAGTTGGGATTCTTGCCTGTG |
| ACCase | TTGGACAAATACGAGAAGGAGGGC | CGCAACTTCCTCGTCAGTCATCAA |
| KAS | CGGCGAACATCAAGGACAAAGAC | CCTGGGAGGTCTTGCCGTCTTT |
| DGAT2 | TCATCTTTGGTAGCCACCCTCAC | CGCCAGCACGCTTCAATCTC |
